# LOGICS: Learning optimal generative distribution for designing de novo chemical structures

**DOI:** 10.1186/s13321-023-00747-3

**Published:** 2023-09-07

**Authors:** Bongsung Bae, Haelee Bae, Hojung Nam

**Affiliations:** 1 School of Electrical Engineering and Computer Science, Gwangju Institute of Science and Technology (GIST), Buk-Gu, Gwangju, 61005 Republic of Korea; 2 AI Graduate School, Gwangju Institute of Science and Technology (GIST), Buk-Gu, Gwangju, 61005 Republic of Korea; 3 Center for AI-Applied High Efficiency Drug Discovery (AHEDD), Gwangju Institute of Science and Technology (GIST), Buk-Gu, Gwangju, 61005 Republic of Korea

**Keywords:** De novo drug design, Deep generative models, Reinforcement learning, Bioactivity optimization

## Abstract

In recent years, the field of computational drug design has made significant strides in the development of artificial intelligence (AI) models for the generation of de novo chemical compounds with desired properties and biological activities, such as enhanced binding affinity to target proteins. These high-affinity compounds have the potential to be developed into more potent therapeutics for a broad spectrum of diseases. Due to the lack of data required for the training of deep generative models, however, some of these approaches have fine-tuned their molecular generators using data obtained from a separate predictor. While these studies show that generative models can produce structures with the desired target properties, it remains unclear whether the diversity of the generated structures and the span of their chemical space align with the distribution of the intended target molecules. In this study, we present a novel generative framework, LOGICS, a framework for Learning Optimal Generative distribution Iteratively for designing target-focused Chemical Structures. We address the exploration—exploitation dilemma, which weighs the choice between exploring new options and exploiting current knowledge. To tackle this issue, we incorporate experience memory and employ a layered tournament selection approach to refine the fine-tuning process. The proposed method was applied to the binding affinity optimization of two target proteins of different protein classes, κ-opioid receptors, and PIK3CA, and the quality and the distribution of the generative molecules were evaluated. The results showed that LOGICS outperforms competing state-of-the-art models and generates more diverse de novo chemical structures with optimized properties. The source code is available at the GitHub repository (https://github.com/GIST-CSBL/LOGICS).

**Supplementary Information:**

The online version contains supplementary material available at 10.1186/s13321-023-00747-3.

## Introduction

Recently, deep-learning techniques have been applied to various aspects of drug discovery [1]. Deep-learning-based approaches are expected to accelerate complex and time-consuming pipelines and reduce the cost of the development of new drugs. The most recent trend in de novo molecular design is the advent of deep generative modeling approaches [2]. Various generative de novo approaches with different neural architectures have been introduced over the past several years [3] (Additional file 1: Section1).

Among a wide spectrum of research, we are especially interested in research on deep generative agents that increase the probability of sampling molecules with desired properties [4–16]. These generative deep networks directly learn the sampling distribution of target molecules. For example, language modeling of simplified molecular-input line-entry system (SMILES) strings produces a model that suggests molecules similar to those in the training set. Note that datasets of known molecules with targeted properties could be too small, and many studies have introduced transfer learning approaches to tackle such low-data problems. Gupta et al. [17] presented the language modeling of SMILES strings using generative long short-term memory (LSTM). They first pre-trained the LSTM on 550,000 SMILES from ChEMBL and fine-tuned the model on specific ligand subsets with known activity for target proteins such as PPARγ, trypsin, and TRPM8. Similar approaches have been employed in other studies [4–6]. Other studies instead utilized the independent prediction model in transfer learning and fine-tuned the generator online without fixing the training data [7–16]. Most of the studies adopted policy-learning reinforcement learning (RL) algorithms by setting the SMILES generation as a sequential decision-making process [7–11], while other studies fine-tuned the maximum-likelihood estimation model by selecting high-scoring molecules from the samples [12–14]. We categorized these methods under the term: generator-predictor collaboration (GPC). A transfer learning method is considered GPC when it meets the following criteria: (a) the fine-tuning dataset is not provided and is not fixed; (b) instead, the fine-tuned dataset is generated by the generator; and (c) the generated samples are evaluated by the separate prediction models to tell the generator whether the samples have positive or negative properties. Previous studies, such as that by Segler et al. [12], REINVENT [7], ReLeaSE [8], DrugEx [9], Augmented Hill-Climb (AHC) [15], and Augmented Memory (AugMem) [16] proposed methods that perfectly fit under the GPC framework, with recurrent neural network (RNN) language models for the generation of SMILES strings.

In GPC studies, the use of the independent prediction model for scoring has been reported to make generative models exploit scores in detrimental ways [14]. In addition, naively training the generator to focus on high-score samples repeatedly often causes a mode collapse of the generator, which is a notorious failure in generative modeling, where the generator only generates samples close to a few target modes and neglects the others [18]. This failure is closely related to the classic explore-exploit dilemma of reinforcement learning, where agents with balanced exploration and exploitation can achieve optimal solutions to complex problems [19]. GPC methods could be susceptible to over-exploitation; thus, a more robust evaluation using independent test sets and distributional metrics is required to detect failures.

To resolve the explore-exploit dilemma of reinforcement learning, we propose LOGICS, a framework for learning the optimal generative distribution iteratively for target-focused chemical structures. LOGICS, a variant of the GPC method, employs experience memory and an advanced selection procedure to approximate the distribution closest to the target chemical space. In this study, the proposed method was demonstrated on the bioactivity optimization towards two protein targets, κ-opioid receptors (KOR) and p110α protein (PIK3CA), and evaluated the distribution of generated molecules using various metrics. We compared LOGICS with other GPC methods from previous studies and performed ablation studies on each component in the framework.

## Materials and methods

### Datasets

The statistics and descriptions of the data used in model construction are summarized in Table 1. We pre-trained the generative LSTM using the SMILES dataset provided by GuacaMol's distribution-learning benchmark [20]. We specified a set of SMILES tokens to be used throughout the study, as described in Additional file 1: Section 2, and removed the SMILES with unspecified tokens in the dataset. Then, each compound's SMILES was canonicalized using RDKit [21] for compound standardization, and duplications were removed.

**Table 1:** Statistics and descriptions of the compound data used in the generator pre-training and the predictor construction

Datasets	Total	Fivefold CV^a^	Test	Active threshold
Pre-training data (Generator)	1,583,425	−	−	−
Training & testing data (Predictor)				
KOR	3881	3230	651	> 7.0 pIC_50_
PIK3CA	1462	1215	247	> 8.0 pKx

^a^Cross-validation

For the target bioactivity predictors, we built activity predictors for each of KOR and PIK3CA. For KOR, the pre-processed bioassay dataset was collected from Pereira et al. [10]. The cited study provided compound activities as pIC_50_ values against KOR, derived from functional assays, accompanied by their SMILES representations. For PIK3CA, we downloaded bioassay data from the PubChem database by querying the PIK3CA target (gene ID:5290) [22]. Notably, we excluded the bioactivity data measured for the mutant form of PIK3CA to maintain data consistency. Also, we filtered the activity data that did not have ' = ' in the 'acqualifier' column. The bioactivity of the compounds for PIK3CA is represented as pKi and pKd, and these two measures are labeled as pKx. Similar to the pre-processing step mentioned above, the compounds were standardized by transforming SMILES into a canonical form. The median value was selected as the representative bioactivity label if a compound had multiple bioactivity values for a given target.

The randomly selected 17% of each bioassay dataset was held out for the test set. The other 83% were used to perform five-fold cross-validation with random splits. After inspecting the label value distribution of each KOR and PIK3CA, the threshold for classifying a molecule to be active was determined to be 7.0 pIC_50_ for KOR and 8.0 pKx for PIK3CA (please see Additional file 1: Section 3). These values were selected such that the numbers of inactive and active molecules were balanced in the data distribution. The active molecules in the test set are referred to as "test set actives" in later sections and were used for evaluating the generative model performance. We suggest the balanced cutoff because if the cutoff is too low (less stringent), all the fine-tuned generators would easily find the active regions, which would diminish the significance of performance differences between the models. Whereas if the cutoff is too high (too stringent), the distributional metrics cannot be appropriately calculated as too few test set actives will be used. While we presume a balanced cutoff is crucial for ensuring a fair comparison of model performance and accurate computation of distributional metrics, the evaluations of generative models using various cutoffs are provided in Additional file 1: Section 4.

## LOGICS framework

- Overview of the proposed approach

**Fig. 1:**
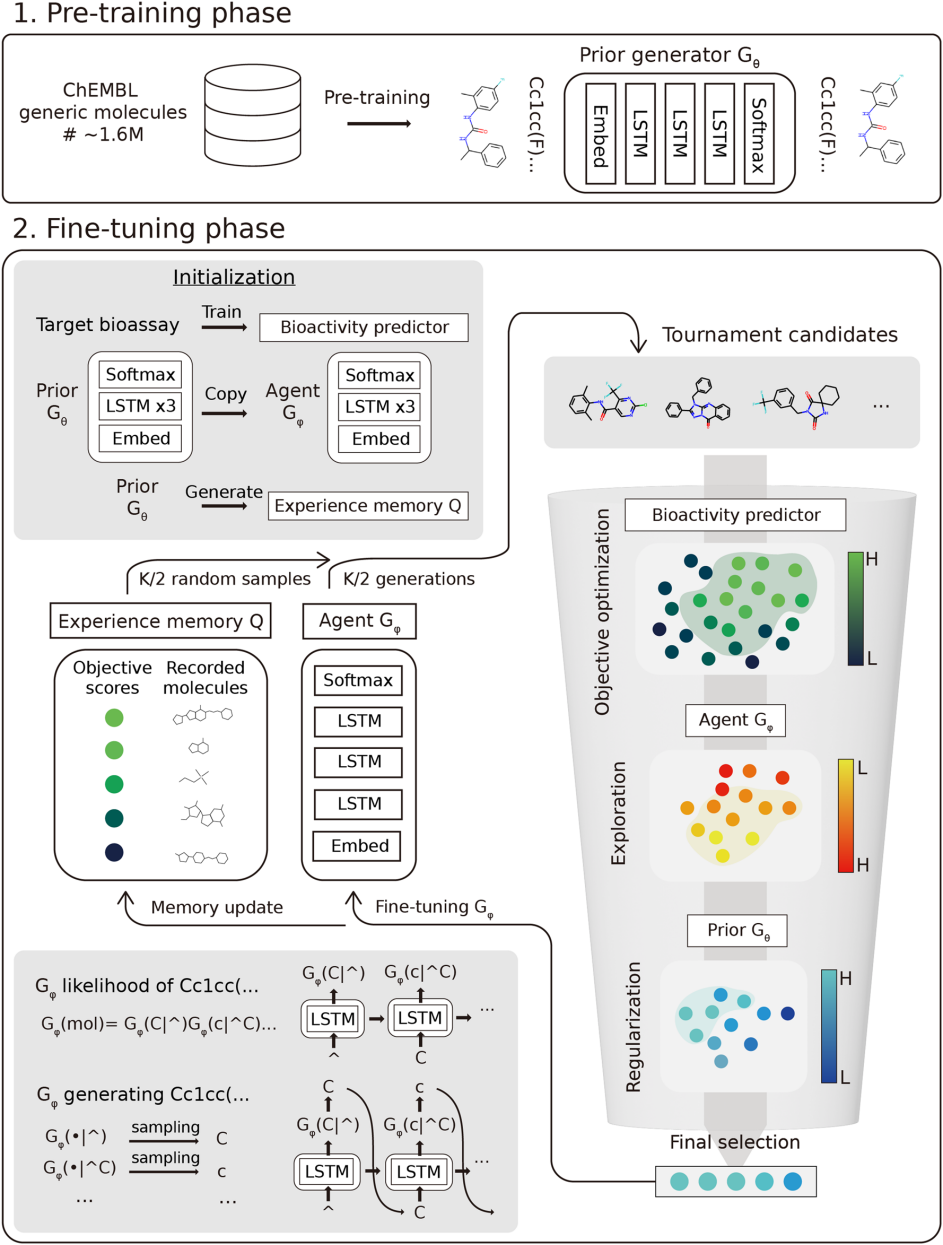
An overview of the proposed LOGICS framework. In the pre-training phase, the prior generator is trained with ~ 1.6 million molecules from ChEMBL. In the fine-tuning phase, the agent generator is fine-tuned with the selected molecules from the generation of the agent itself and the experience memory, where the selection is performed with the three stages of tournament selections

The LOGICS framework proposed in this study follows the GPC scheme (Fig. 1). The binding affinity prediction model, *predictor*, was trained with the pre-processed bioassay dataset described in the Datasets section and used to signal the rewards that guide the fine-tuning of the generators. For the feature vectors of the regression models, RDKit's Morgan fingerprint using 2048 bits and a radius of 2 was used. Random forest regressors (RFR) were trained using five-fold cross-validation.

The SMILES generation model used in this study is a generative RNN for sequence modeling similar to those introduced in previous studies [7–10, 12, 23–25]. The teacher forcing method was adopted to train the sequence model [25], and the negative log-likelihood (NLL) for the given training sequence, $$\begin{aligned} l(\theta) &=-{\sum }_{t=1}^{T}log({G}_{\theta}({x}_{t}|{x}_{0:t-1}))\\ &=-log{\prod}_{t=1}^{T}{G}_{\theta}({x}_{t}|{x}_{0:t-1}), \end{aligned}$$ was minimized using the Adam optimizer. We refer to the pre-trained generator as the prior generator (*G*_*θ*_). The pre-trained generative LSTMs can be used not only for generating random molecules with ChEMBL-like properties but also for measuring the probability of sampling a given SMILES sequence by the LSTM as follows: ${G}_{\theta }({x}_{1:T})={\prod }_{t=1}^{T}{G}_{\theta }({x}_{t}|{x}_{0:t-1}).$

We refer to this probability of generating a given sequence using ${G}_{\theta }$ as the *prior likelihood*, following the terminology used in a previous report [7]. Refer to Additional file 1: Section 5 for more details on the predictor and prior generator. The main contribution of the proposed work resides in the fine-tuning step, and the details are described in the following subsection.

The performance of the model was evaluated based on various metrics, such as validity, uniqueness, and the average predicted activity of the generations. In addition to these metrics, we compared the distributions of the generated molecular space and the unseen target active molecular space. The Fréchet ChemNet distance (FCD) [26] and optimal transport distance (OTD) between generations and test set actives were evaluated, and 2-D t-distributed stochastic neighbor embedding (t-SNE) visualizations were performed accordingly. Refer to the Evaluation metrics subsection for further details.

b. Fine-tuning and tournament selection

Our fine-tuning algorithm introduces a few components. *G*_*φ*_ is an agent generator, which is a generative LSTM being trained through the fine-tuning phase. *Q* is an experience memory, which stores the generated molecules that survived through the tournament selections. The fine-tuning phase begins with agent generator initialization with prior model weights, and initialization of *Q* with the *P* unique molecules generated by *G*_*θ*_ (Algorithm 1). We can calculate the probability of sampling a given sequence ${x}_{1:T}$ from an agent, referred to as *agent likelihood*, as follows: ${G}_{\varphi }({x}_{1:T})={\prod }_{t=1}^{T}{G}_{\varphi }({x}_{t}|{x}_{0:t-1}).$

In the fine-tuning loop, we attempt to create an optimal synthetic training set that can guide the agent towards high-score regions with the right balance between exploration and exploitation. The first step is to form initial candidates of size *K*. The initial candidates were formed by *K*/2 random samples from the memory and *K*/2 generations from the agent. These candidates then go through a three-stage tournament selection procedure (Algorithm 2). Each tournament was used to select individuals with a high value of the given scoring function for the stage. The first-stage tournament, referred to as the *objective stage*, is performed by the objective function; in our case, the predicted bioactivity. The second stage, the *exploration stage,* is performed using the negative agent likelihood. Finally, the third stage, the *regularization stage*, is performed using the positive prior likelihood. While solely focusing on maximizing the objective, it can lead to a low-exploration challenge, as noted in the Introduction. Thus, the exploration stage is adopted to select molecules that are unlikely to be generated by the current agent. The role of the regularization stage is to prevent catastrophic forgetting, where the agent forgets the basic chemical grammar learned in the pre-training phase by dropping molecules too far from the prior distribution. After the three-stage tournament, *W*, the set of finally selected molecules, is obtained and replaces the low-scoring individuals in memory *Q*. Then, agent *G*_*φ*_ is fine-tuned with *W*.
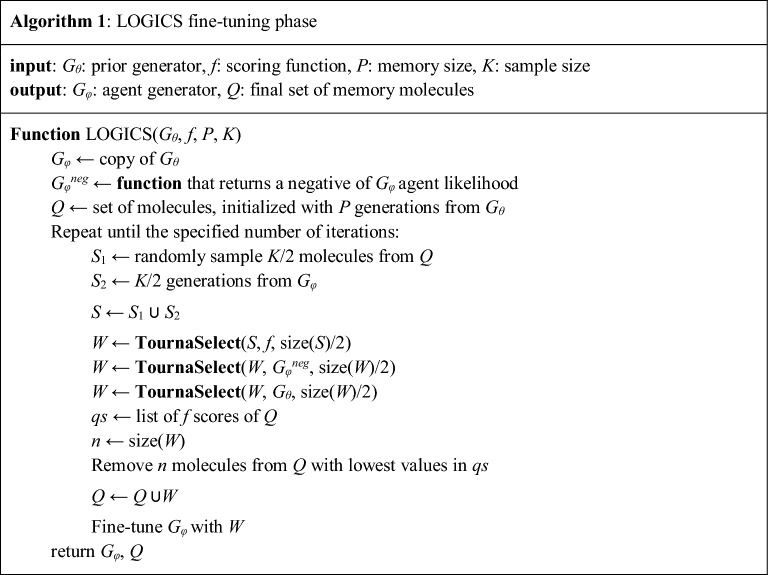



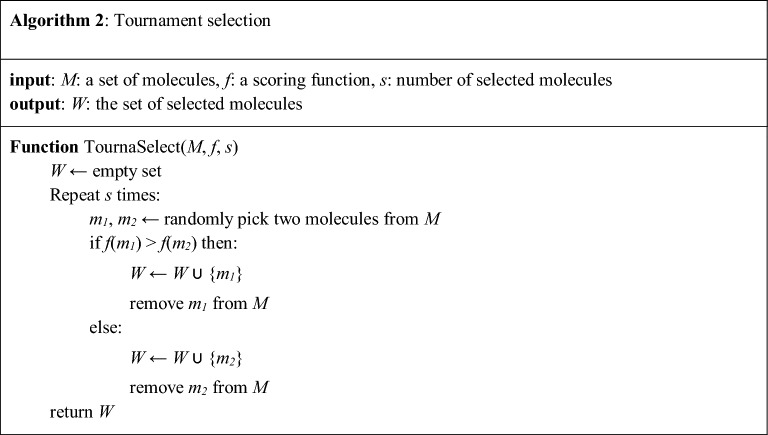


The filtering procedure in the loop should take into account various factors to promote the exploration of chemical space, including the exploration stage of tournaments. Tournament selection is adopted to resolve the early overfitting problem of the Vanilla GPC (VGPC) model, as it only selects the top *n* best generations for the next training set. Tournament selection promotes randomness in that there is a small chance for some inferior candidates to survive [27]. This property of tournament selection encourages exploration by the agent. Another factor is the use of experience memory. We presume that the mode-collapsing behaviors of the GPC models in late-stage fine-tuning can be mitigated by providing them with good samples generated in the previous training stages. Experience memory is adopted to record the various sound generation examples during the fine-tuning phase, so that the agent, which has found a small collapse point of the high-score region, can escape from collapse by remembering the previous experience. This concept is similar to replay buffer mechanisms [28], which are now a staple component of most state-of-the-art reinforcement learning algorithms.

## Evaluation metrics

In this study, we used two types of evaluation metrics: (1) standard metrics and (2) optimization metrics. First, for the standard metrics, *N* samples were generated as representatives of the generative distribution of the model for each training epoch. In the experiments, we set *N* = 20,000. *V* is the set of valid generations among *N*, *U* is the set of unique molecules among *V*, and *Z* is the set of molecules that are not found in the pre-training dataset among *U*. The standard metrics are similar to those introduced in previous studies [20, 29].

$$Validity=|V|/N$$

$$Uniqueness=|U|/|V|$$

$$Novelty=|Z|/|U|$$

$$Internal\,Diversity=\frac{1}{{|{V}_{1k}|}^{2}}{\sum }_{{m}_{1}\in {V}_{1k}, {m}_{2}\in {V}_{1k}}1-sim({m}_{1},{m}_{2})$$

where *sim*(*m*_*1*_*,m*_*2*_) is the Tanimoto similarity of Morgan fingerprint vectors of 2048 bits and a radius of 2 between molecules *a* and *b*. *V*_*1k*_ is a random subset of 1000 from *V.* Smaller generation sets are used to calculate the pairwise similarities because of the high computational costs. The standard metrics are used to evaluate the capability of chemical generative models to determine whether the model can generate valid and diverse molecules. These metrics are calculated purely based on the model's generation, not factoring in the generation's objective scores or closeness to the target distribution.

For the optimization metrics, we used the average of the predicted bioactivity (PredAct), the average of pairwise similarity between generations and test set actives (PwSim), FCD, and OTD. Higher values of PredAct and PwSim and lower values of FCD and OTD are desired for generative models. In this study, we use FCD to measure the distance between the distribution of test set actives and the distribution of model generations. In the following equations, *T* represents the set of target active molecules in the test or validation set. PwSim and FCD were calculated as follows:

$$PwSim=\frac{1}{|{V}_{1k}||T|}{\sum }_{{m}_{1}\in {V}_{1k}, {m}_{2}\in T}sim({m}_{1},{m}_{2})$$

$$FCD=||{\mu }_{V}-{\mu }_{T}{||}^{2}+Tr({C}_{V}+{C}_{T}-{2({C}_{V}{C}_{T})}^{1/2})$$

where ${\mu }_{V}$ and ${\mu }_{T}$ are the means of the feature vectors generated by the ChemNet model from a previous study [26] using *V* and *T*, while ${C}_{V}$ and ${C}_{T}$ are the covariances of the vectors.

FCD assumes that the two point sets *V* and *T* are normally distributed [26]. This assumption is not ideal if the target distribution formed by *T* consists of multiple modes. Considering that two structurally different molecules could show similar binding affinities for a protein target [30], it is reasonable to presume the target distribution had multiple modes in our experiments. Thus, we propose the use of another distributional metric, OTD. In the theory of optimal transport, the distance between two probability distributions can be measured by determining the transport plan between the two sets of masses, thus minimizing the cost of the total transportation [31]. Based on the concepts from discrete optimal transports [32], we determine the optimal transport *T* by solving the following equation:

$${argmin}_{T\in R}{\sum }_{{x}_{i}\in A, {y}_{j}\in B}{T}_{ij} dist({x}_{i},{y}_{j})$$ s.t. $${T}_{ij}\in \{0,\frac{1}{c}\},{\sum }_{{y}_{j}\in B}{T}_{ij}=\frac{1}{c},{\sum }_{{x}_{i}\in A}{T}_{ij}=\frac{1}{c}$$

where ${T}_{ij}$ is the transport mass from point ${x}_{i}$ to the point ${y}_{j}$ and *R* is the set of all possible one-to-one mappings from *A* to *B* [33]. In our experiments, *A* is the set of generated molecules and *B* is the set of test set actives. The distance used for OTD calculation is: $dist({x}_{i},{y}_{j})={10}^{1-sim({x}_{i},{y}_{j})}-1.$

Further details on the metrics are in Additional file 1: Section 6.

## Results and discussion

### Performance of the pre-trained model and bioactive predictor

The pre-trained generative LSTM, or prior model, achieved 0.9536 validity, 0.9992 uniqueness, 0.9483 novelty, and 0.8894 diversity, which are similar values to the reported performance of GuacaMol [20]. For bioactive prediction, the MSE and R^2^ were 0.5416 and 0.7132 for KOR and 0.4867 and 0.7594 for PIK3CA, respectively. The detailed performance of the predictors is presented in of Additional file 1: Table S2 and Figure S4 in Section 7.

## Performance of the fine-tuned generator

To evaluate the performance of the proposed framework LOGICS, we performed a comprehensive analysis using various metrics and compared it to other related GPC approaches, including VGPC, Segler [12], REINVENT [7], DrugEx [9], AHC [15], and AugMem [16]; refer to Additional file 1: Section 8 for more details. For a fair comparison, all approaches were evaluated under identical conditions, i.e., starting from the same prior generator and using the same bioactivity predictors. Using the same prior model and predictors, each GPC method uses a different fine-tuning algorithm to produce an optimized agent generator. The reward signal comes solely from the predictors, and no information about the known active molecules is provided directly. For policy gradient methods, such as REINVENT, DrugEx, AHC, and AugMem, the transformation from predicted bioactivity to the reward value was applied to stabilize the training process, as described in Additional file 1: Section 8-2. We provided the models with arbitrarily large numbers of epochs to make sure that each model converges. We then selected the best stopping epoch for the evaluation of each method under two conditions: the model should (1) achieve a PredAct higher than the target activity threshold at the selected epoch and (2) have the minimum OTD × FCD value for the target actives from the validation dataset. Once fine-tuning ceased at the chosen epoch, we generated 20,000 SMILES samples from each model and computed the evaluation metrics using these samples. Each model underwent evaluation three times, and we reported the mean and standard error of the mean for the three values across each metric (Table 2).

**Table 2:** Comparison of bioactivity optimization performance for KOR and PIK3CA with various GPC methods

	Prior	VGPC	Segler	REINVENT	DrugEx	AHC	AugMem	LOGICS
KOR								
Validity^a,α^	0.95 ± 0.00	0.93 ± 0.02	0.96 ± 0.00	0.98 ± 0.00	0.97 ± 0.00	0.97 ± 0.00	0.96 ± 0.00	0.98 ± 0.00
Unique^b,α^	0.99 ± 0.00	0.99 ± 0.00	0.94 ± 0.00	0.90 ± 0.00	0.99 ± 0.00	0.87 ± 0.00	0.96 ± 0.00	0.99 ± 0.00
Novelty^c,α^	0.94 ± 0.00	0.99 ± 0.00	0.99 ± 0.00	0.94 ± 0.00	0.99 ± 0.00	0.95 ± 0.00	0.97 ± 0.00	0.98 ± 0.00
Diversity^d,α^	0.88 ± 0.00	0.79 ± 0.00	0.85 ± 0.00	0.86 ± 0.00	0.83 ± 0.00	0.86 ± 0.00	0.86 ± 0.00	0.85 ± 0.00
PredAct^e,β^	5.95 ± 0.00	7.04 ± 0.00	**8.30 ± 0.00**	7.04 ± 0.01	7.10 ± 0.00	7.16 ± 0.00	7.00 ± 0.00	7.57 ± 0.00
PwSim^f,β^	0.11 ± 0.00	0.10 ± 0.00	0.12 ± 0.00	0.12 ± 0.00	0.12 ± 0.00	0.12 ± 0.00	0.12 ± 0.00	**0.13 ± 0.00**
FCD^g,β^	27.2 ± 0.02	38.8 ± 0.06	22.3 ± 0.03	26.0 ± 0.06	30.4 ± 0.06	24.6 ± 0.05	26.0 ± 0.06	**22.2 ± 0.01**
OTD^h,β^	5.37 ± 0.00	5.85 ± 0.00	5.09 ± 0.00	5.11 ± 0.00	5.37 ± 0.00	5.23 ± 0.00	5.27 ± 0.00	**4.95 ± 0.00**
PIK3CA								
Validity^a,α^	0.95 ± 0.00	0.85 ± 0.00	0.97 ± 0.00	0.99 ± 0.00	0.98 ± 0.00	0.97 ± 0.00	0.98 ± 0.00	0.99 ± 0.00
Unique^b,α^	0.99 ± 0.00	0.99 ± 0.00	0.94 ± 0.00	0.65 ± 0.00	0.99 ± 0.00	0.91 ± 0.00	0.92 ± 0.00	0.71 ± 0.00
Novelty^c,α^	0.94 ± 0.00	0.99 ± 0.00	0.99 ± 0.00	0.93 ± 0.00	0.99 ± 0.00	0.96 ± 0.00	0.97 ± 0.00	0.99 ± 0.00
Diversity^d,α^	0.88 ± 0.00	0.82 ± 0.00	0.78 ± 0.00	0.79 ± 0.00	0.80 ± 0.00	0.82 ± 0.00	0.81 ± 0.00	0.73 ± 0.00
PredAct^e,β^	6.84 ± 0.00	8.05 ± 0.00	8.75 ± 0.00	8.83 ± 0.00	8.39 ± 0.00	8.01 ± 0.00	7.99 ± 0.00	**9.54 ± 0.00**
PwSim^f,β^	0.10 ± 0.00	0.11 ± 0.00	0.11 ± 0.00	0.17 ± 0.00	0.11 ± 0.00	0.10 ± 0.00	0.10 ± 0.00	**0.18 ± 0.00**
FCD^g,β^	41.0 ± 0.08	43.7 ± 0.06	45.7 ± 0.02	32.7 ± 0.08	44.1 ± 0.11	51.0 ± 0.07	50.9 ± 0.03	**29.4 ± 0.10**
OTD^h,β^	5.99 ± 0.01	5.93 ± 0.00	5.78 ± 0.01	4.47 ± 0.02	5.88 ± 0.01	5.94 ± 0.00	5.97 ± 0.00	**4.27 ± 0.02**

Bold represents the best-performing value among the methods

^a^Validity is the ratio of valid generations to 20,000 generations from the model

^b^Uniqueness is the ratio of unique generations to the valid generations

^c^Novelty is the ratio of unique generations that are not found in the pre-training dataset

^d^Diversity measures how dissimilar the 1,000 generations are

^e^PredAct is the mean of predicted activities of the valid generations

^f^PwSim is the mean of pairwise similarities between generations and test set activities

^g^FCD is the Fréchet Chemnet Distance between the generations and the test set activities

^h^OTD is the optimal transport distance between generations and the test set activities

^α^Standard metrics

^β^Optimization metrics

**Fig. 2:**
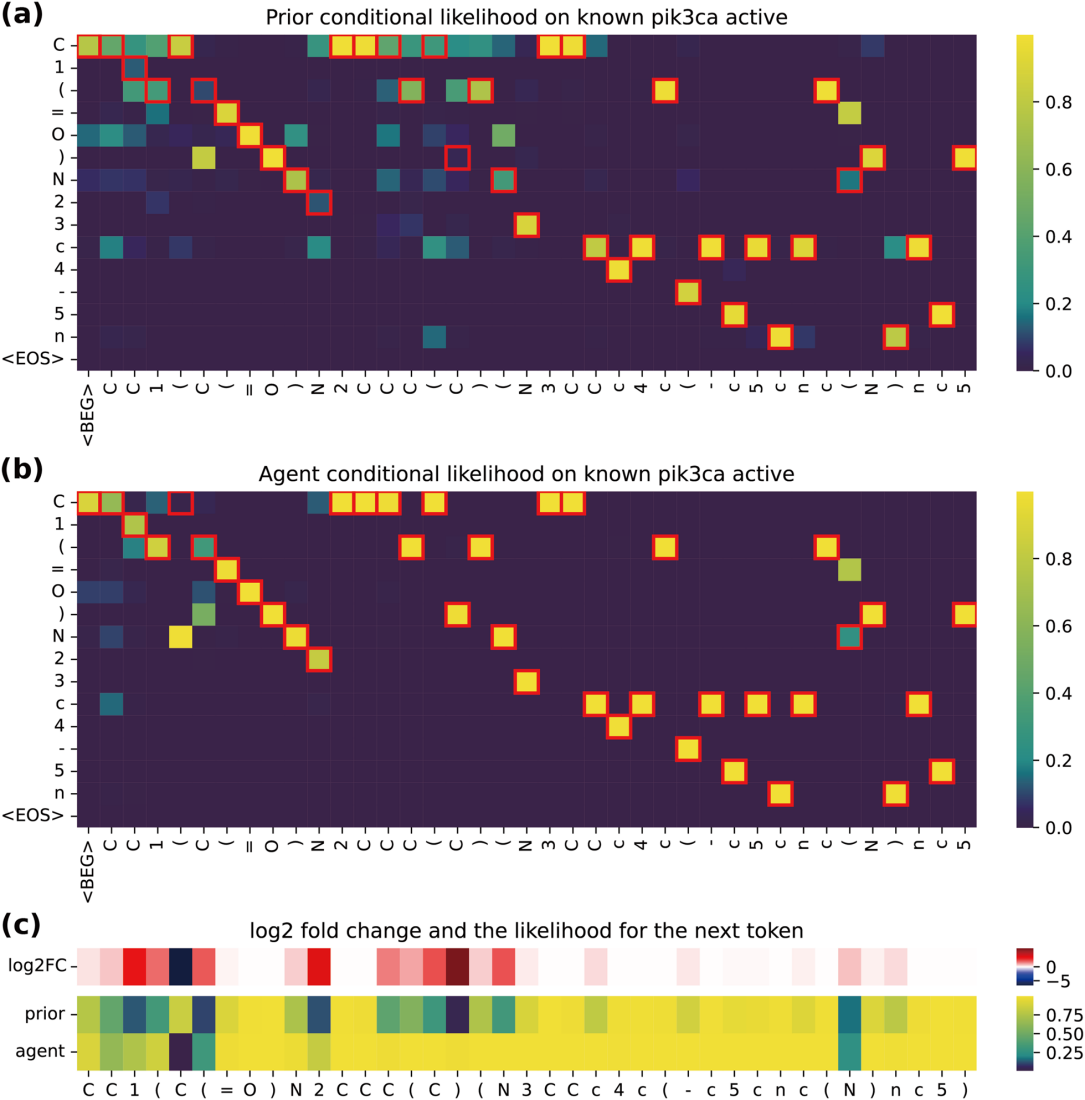
Heatmap visualization of LSTM softmax probability outputs on a known PIK3CA active. We used a known PIK3CA active, “CC1(C(= O)N2CCCI(N3CCc4c(-c5cnc(N)nc5)nc(N5CCOCC5)nc43)C2)CCCO1” with 10.2 pKd activity recorded for PIK3CA. The given SMILES is cut off at the first 40 tokens for the visualization. **a**, **b** The tokens on the x-axis are the input SMILES on the time steps, and the token on the y-axis corresponds to each output of the softmax layer of the prior generator and the agent generator, respectively. The highlighted cells indicate the correct next tokens to be sampled to obtain the given sequence. **a** The prior model's conditional likelihood heatmap. **b** The conditional likelihood heatmap of the LOGICS agent from the PIK3CA experiment. **c** Side-by-side comparison of the correct next token output probability of the prior and the agent and the log2 fold change of the agent likelihood over the prior likelihood. The fold change is calculated by ${log}_{2}{G}_{\varphi }({x}_{t}|{x}_{0:t-1})/{G}_{\theta }({x}_{t}|{x}_{0:t-1})$

Table 2 shows the performance of each method for KOR and PIK3CA bioactivity optimization. Additional file 1: Figures S6 and S7 in Section 9 show the change in each performance metric according to the training progress in the fine-tuning phase for KOR and PIK3CA, respectively. Table 2 demonstrates that all methods exhibit a competitive performance according to the standard evaluation metrics, such as validity and uniqueness. According to the optimization metrics in Table 2, LOGICS demonstrated the best overall performance. It achieved the minimum FCD and OTD values, indicating that the proposed method learned the closest generative distribution to the test set actives. We also confirmed the FCD and OTD performances achieved by LOGICS are significantly better than the other methods in statistical tests, as shown in Additional file 1: Table S4 in Section 10. Figure 2 shows the effect of the fine-tuning phase of the LOGICS framework. We selected a molecule with known PIK3CA activity from the bioassay data and visualized the conditional probability output from the agent LSTM. Compared to Fig. 2A, the heatmap in Fig. 2B shows that certain tokens have a higher probability of being generated. This result indicates that the fine-tuned agent is more likely to generate SMILES with a higher activity level.

In comparison to the prior model's performance (Table 2), most of the methods were able to achieve a better PredAct, which indicates the potential of optimized bioactivity. Their uniqueness and diversity also remained fairly high. However, some models, such as VGPC and DrugEx, performed worse than the prior model in terms of the FCD and OTD. This indicates that these models only learned to generate a particular portion of the target active molecular space with high predicted scores and were incapable of discovering other spaces with known target activity. These results reconfirm that the GPC method is susceptible to mode collapse and catastrophic forgetting [34, 35]. In VGPC, we consider that these failures occur because of the repeated maximization of the probability of high-score molecules generated early in the fine-tuning phase. Once the probability of a certain high-score molecule is maximized, the next iteration would be more likely to generate similar structures to the molecule with the learned patterns. Repeated maximization in the loop eventually leads to a mode-collapsed generative distribution. Additional file 1: Figures S6 and S7 further support our result, as the constant increase in PredAct in the fine-tuning process does not directly correspond to the decrease in OTD and FCD in many of the tested models. Moreover, we note that the proposed selection procedure in LOGICS appears to be more effective than the other exploration strategies employed by DrugEx, as LOGICS achieved higher optimization metric scores than DrugEx.

Additionally, to achieve lower OTD and FCD values, the algorithm needs to employ a bit of the exploitation strategy because focusing only on exploration can lead to a diverging distribution that never converges. Therefore, a balance between exploration and exploitation is required. Indeed, the models with low uniqueness, i.e., REINVENT and LOGICS, showed better OTD and FCD values than the other models, which indicates that the two methods could converge to the various target active spaces with the right balance of exploitation and exploration. In the PIK3CA experiment, AHC and AugMem performed significantly worse OTD and FCD compared to the other methods, and they notably deteriorated from REINVENT, even though they are the extensions of REINVENT. We presume the degradation of learned distribution comes from the application of the diversity filter (DF) of AHC and AugMem. The DF could penalize the highly active molecules encountered early in the iterations, and the molecules cannot be repeatedly sampled, which is essential for learning the optimal distribution that covers as many active regions as possible. In other words, the DFs forced too much exploration in AHC and AugMem, and thus, appropriate exploitation cannot occur during the fine-tuning.

We also verified the robustness of the LOGICS framework to the training data size and structural diversity. The detailed discussion can be found in Additional file 1: Section 11. Moreover, we found one of the practical benefits of LOGICS is that it doesn't require reward function engineering, unlike most RL-based policy gradient models. The discussion on this topic is detailed in Additional file 1: Section 12.

## Distribution of generated molecules in chemical space

**Fig. 3:**
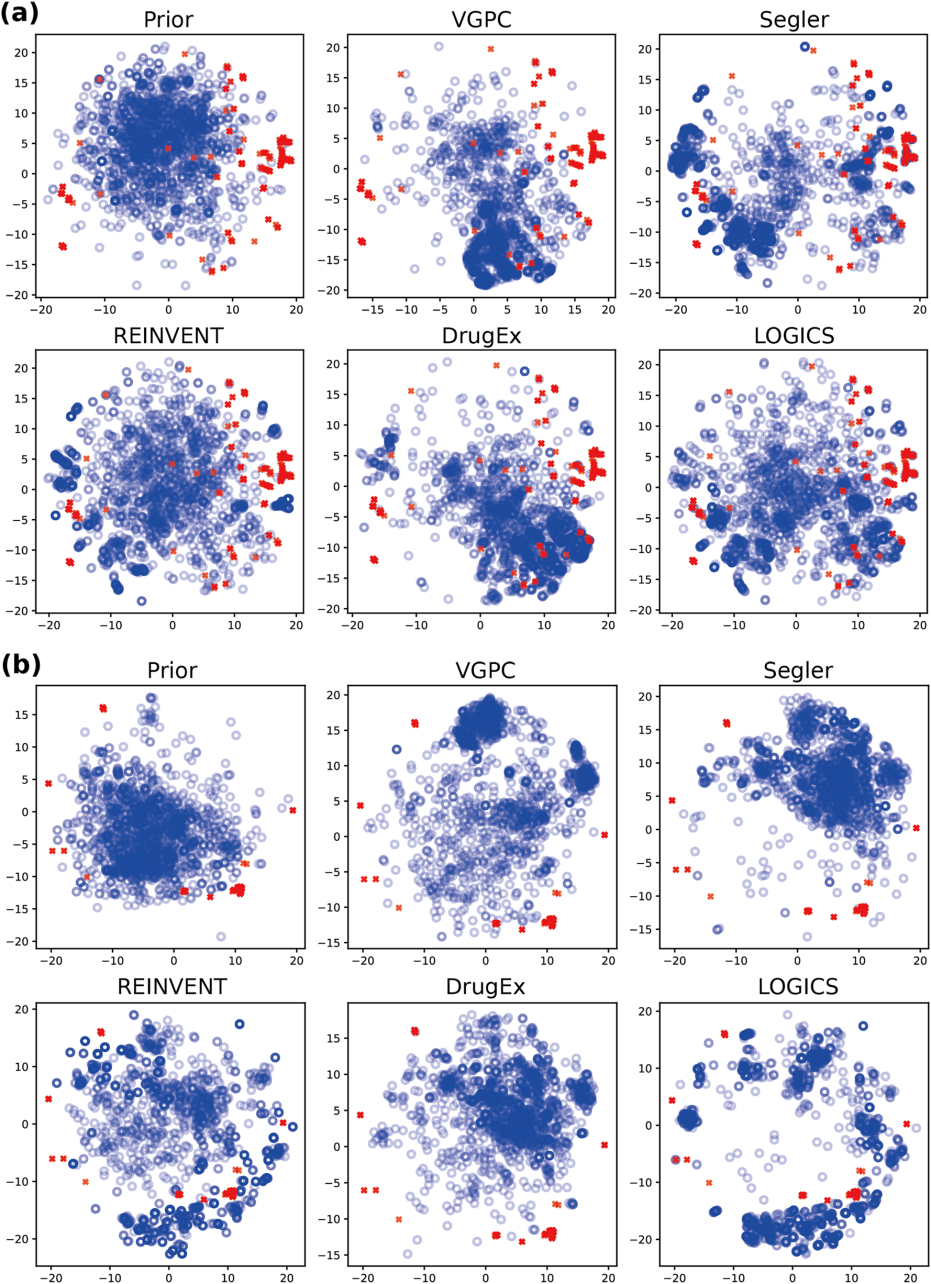
Chemical space visualization by t-SNE. Chemical space visualization of the Morgan fingerprint vectors transformed by t-SNE on (**a**) the KOR experiment and (**b**) the PIK3CA experiment, respectively. The t-SNE components were calculated with the test set actives (red) and the valid molecules among generations from the prior model as well as the different GPC models (blue). For the purpose of visualization, 2,000 randomly selected, valid, generated molecules were used for each generative model

Figure 3 shows the generative distribution of each fine-tuned generator in the KOR and PIK3CA bioactivity optimization experiments. We collected 20,000 valid generations from each model, including the prior, 50,000 random molecules from the pre-training data, and test set actives. We derived the Morgan fingerprint vectors, and transformed the vectors to the 2-D space using t-SNE as described in [36] with default parameters.

Figure 3 demonstrates that the prior distributions could not generate most of the test set actives. According to Fig. 3A, the LOGICS, REINVENT, and Segler methods discovered most of the KOR test active regions, whereas the other two methods, VGPC and DrugEx, could not fully explore enough to cover the test set actives. This visual result corresponds to the analysis from the previous section in Table 2, where the OTD and FCD of the VGPC and DrugEx were not better than the prior. We can also infer the effectiveness of the distribution metrics based on these results. In Fig. 3A, DrugEx seems to generate a few molecules close to the test set actives at the (−16, −4) and (17, 3) coordinates; however, most of its generations are concentrated at the (10, −10) coordinates. From the perspective of distribution learning, DrugEx's generative distribution is biased toward only a few target active regions; thus, it should show worse scores of the distributional metrics, as shown in Table 2. The difference between the learned distributions of the GPC methods is more prominent in Fig. 3B. In the figure, LOGICS and REINVENT could properly bias the distributions towards the regions of the test set actives, while the other methods focused on the regions that did not include any test set actives. This also corresponds with the numerical data in Table 2, where the models with a distribution similar to the test set actives achieved far better optimization scores. In addition, by inspecting the figure, we can see why LOGICS achieved better optimization scores than REINVENT. LOGICS exhibited superior exploitation behavior than REINVENT in that LOGICS completely abandoned the prior distribution and focused more on the spaces close to the test set actives.

## Ablation study

**Fig. 4:**
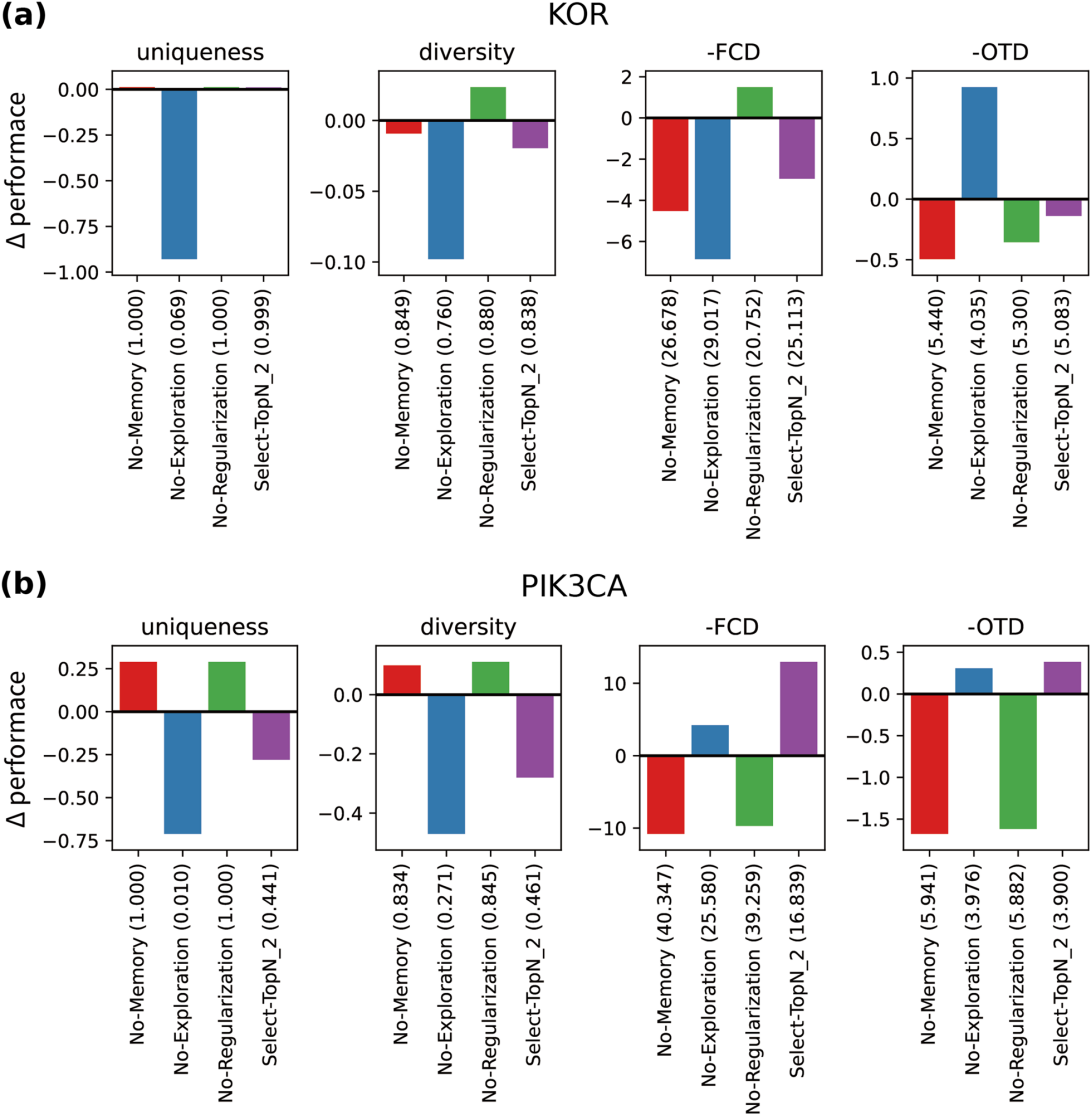
Ablation study. Ablation study of LOGICS framework on (**a**) the KOR bioactivity optimization, and (**b**) the PIK3CA bioactivity optimization. Four significant metrics are reported: uniqueness, diversity, FCD, and OTD. Four unique models (x-axis) with a specific disabled component are tested. The y-axis represents the model's performance minus the full LOGICS model performance value. For the FCD and OTD, the negative value of the score is used so that a bar below 0 represents the worse performance of the disabled model. The actual performance value of each model is shown in the parentheses of the model name on the x-axis

To examine the impact of the individual LOGICS framework components on the performance of generative modeling, we conducted ablation studies. We constructed four ablation models: (i) removal of the memory (*no-memory*), (ii) removal of the exploration stage (*no-exploration*), (iii) removal of the regularization stage (*no-regularization*), and (iv) substitution of tournament selection with another selection method and choosing candidates with scores above the median (*select-topN/2*). We tested four disabled models for KOR and PIK3CA bioactivity optimization. The performance difference between LOGICS and the disabled models is shown in Fig. 4. The exact performances are described in Additional file 1: Table S8 in Section 13. In Fig. 4, the four significant performance metrics, uniqueness, diversity, FCD, and OTD, are selected to demonstrate the performance differences. The bars in the uniqueness and diversity charts represent the metric value of the disabled model minus that of the full LOGICS model. The bars in the FCD and OTD charts represent the metric value of the full model minus that of the disabled model. Thus, for all metrics, a value below zero represents performance degradation.

We confirmed the importance of the exploration stage (Fig. 4). The uniqueness and diversity of the no-exploration model showed a significant drop in performance. Specifically, this model exhibited 0.01 uniqueness in the PIK3CA case, which means that 99% of its valid generations were duplicates, and 0.271 diversity, which is also very low, considering that diversity of the other models presented in this study never fell below 0.3 in Additional file 1: Figure S7. The 4.035 OTD in the KOR case appears to be an improvement in the distance to the target, but the FCD was the worst of all the models, as shown in Fig. 4A. A discrepancy between the OTD and FCD is possible when the duplicated generations of the no-exploration model are located in the middle of a cluster of test set actives. As a result, the OTD can be decreased suboptimally, while the FCD is penalized with a very low variance in the generated distribution. This discrepancy between OTD and FCD (Fig. 4A) exemplifies the importance of inspecting various performance metrics in evaluating generative models, as using a single metric, OTD in this case, to evaluate a model's capability could be misleading.

**Fig. 5:**
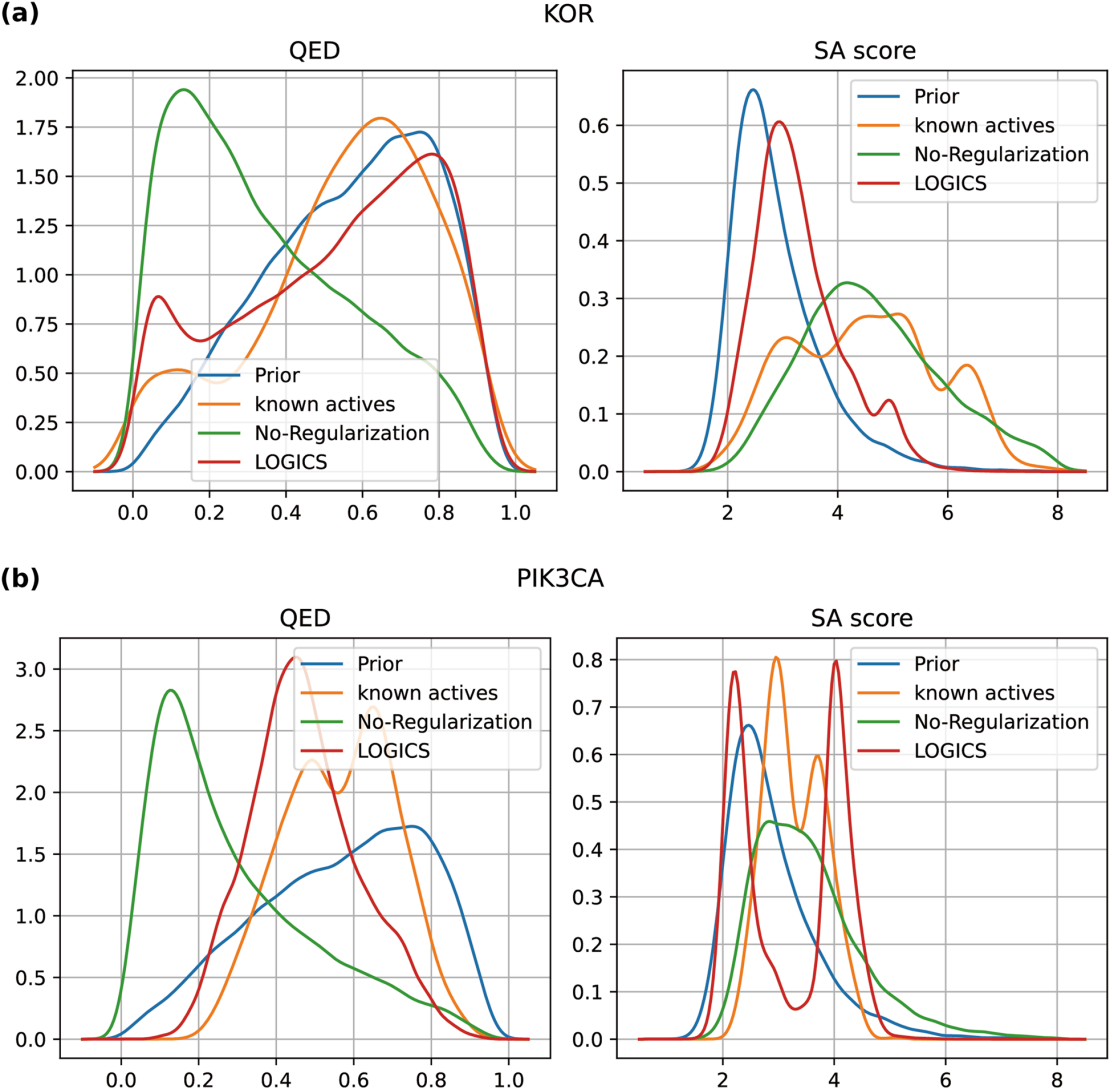
QED and SA score. QED and SA score density plots of generated molecules from the prior, no-regularization, and LOGICS models and the known actives from (**a**) the KOR and (**b**) PIK3CA experiments. QED and SA score were calculated using RDKit

Disabling the regularization stage in the no-regularization model deteriorated the FCD and OTD in the PIK3CA case (Fig. 4B). Although the no-regularization model did not show much difference in performance in the KOR case (Fig. 4A**)**, the quality of the generations decreased (Fig. 5A**)**. We calculated the quantitative estimate of drug-likeness (QED) and synthetic accessibility (SA) scores of the generated molecules using modules provided by RDKit [21]. A higher QED implies greater drug-likeness of the generated molecule [37], whereas a lower SA score indicates that the molecule is easier to synthesize. [38]. The no-regularization model with lower QEDs is compared to the full LOGICS model in both the KOR and PIK3CA experiments (Fig. 5). In the KOR case, the SA scores of the no-regularization model were also significantly higher (less synthesizable) than those of the full model (Fig. 5A). On the basis of these results, we can conclude that the regularization stage serves to preserve the chemical quality of the generative distribution learned in the pre-training phase.

The select-topN/2 model showed an improved distribution closer to the PIK3CA test set actives (Fig. 4B) according to the FCD and OTD values, while it showed a worse distribution that was far from the KOR test set actives (Fig. 4A). However, the closer distance to the PIK3CA test set actives was achieved at the cost of uniqueness and diversity. Thus, disabling the exploration ability of tournament selection harbors trade-offs.

Figure 4 shows that disabling experience memory reduces FCD and OTD values the most. This is to be expected, as the memory contains good examples that survived the three tournament stages in previous iterations, combining exploitation, exploration, and regularization. The absence of memory diminishes the overall performance of the framework.

Based on the ablation studies, we presume that the experience memory and selection mechanisms could benefit other GPC methods that struggle to learn the optimal distribution. We modified a GPC method called ReLeaSE from a previous study [8], by adding the LOGICS components in the fine-tuning phase, and evaluated the performance of the model before and after the modification (Additional file 1: Section 14).

## Examples of generated de novo structures

**Fig. 6:**
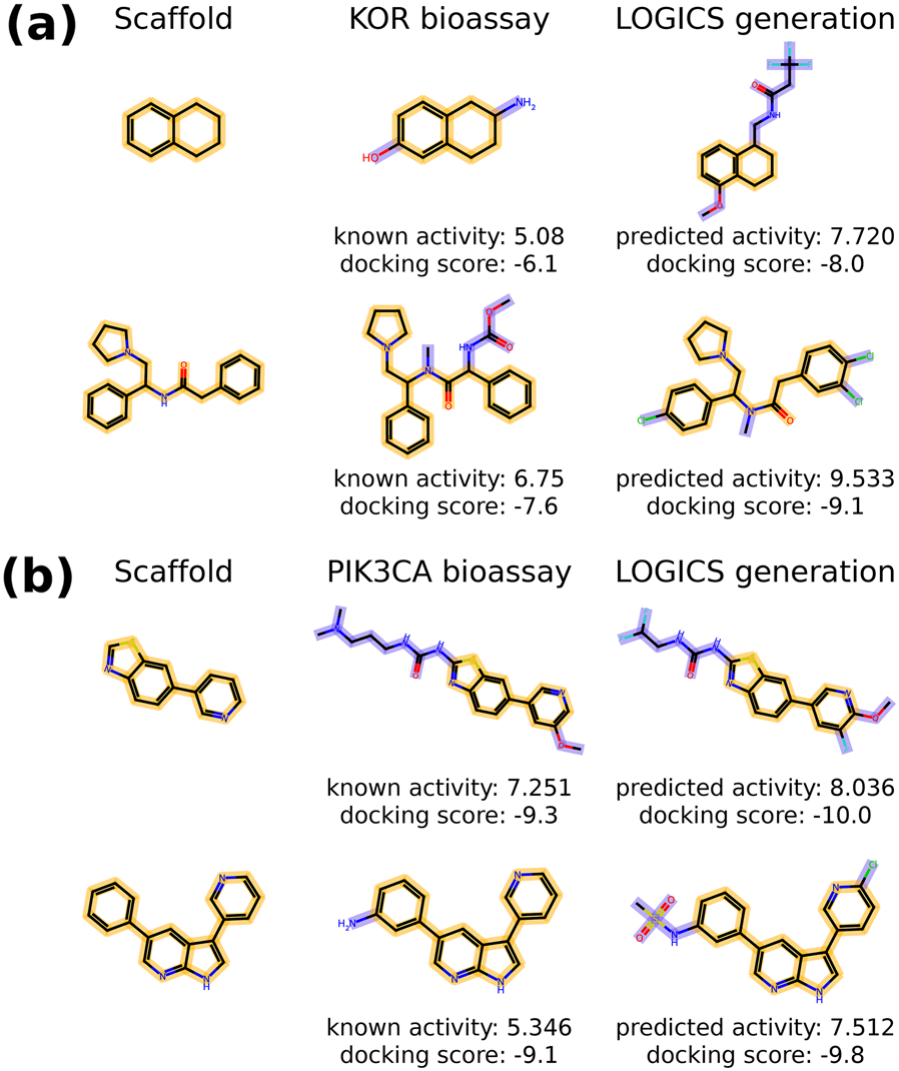
Generated molecules sharing the same scaffold as the molecules from the bioassay. Generated molecules in the 2-D structure by LOGICS where the generations and the molecules from the bioassay data share the same scaffold. **a** The scaffold is from the KOR bioassay. **b** The scaffold is from the PIK3CA bioassay

Here, we illustrate examples of de novo structures generated by the fine-tuned LOGICS model from the KOR and PIK3CA experiments. These results demonstrate that LOGICS can be applied in either direction to generate de novo compounds for optimizing existing scaffolds or to generate novel scaffolds for expanding potent structures. Figure 6 illustrates the generated molecules that share the same scaffold as the molecule in the bioassay data. In each instance, LOGICS was able to generate a structure with higher activity than the previously identified molecule containing the same scaffold. The generated molecules also retain better docking scores to the target protein structures compared to the reference molecules. This preferable docking of optimized generation is not only specific to the case in Fig. 6, but we also confirmed that the docking scores of LOGICS generation are significantly better in general compared to the prior generator, as shown in Additional file 1: Figure S8 (refer to the detailed results in Additional file 1: Section 15). Additionally, we performed the retrosynthetic prediction for the generated molecules in Fig. 6, and all of the four generated molecules are found to be synthesizable as shown in of Additional file 1: Section 16.

**Fig. 7:**
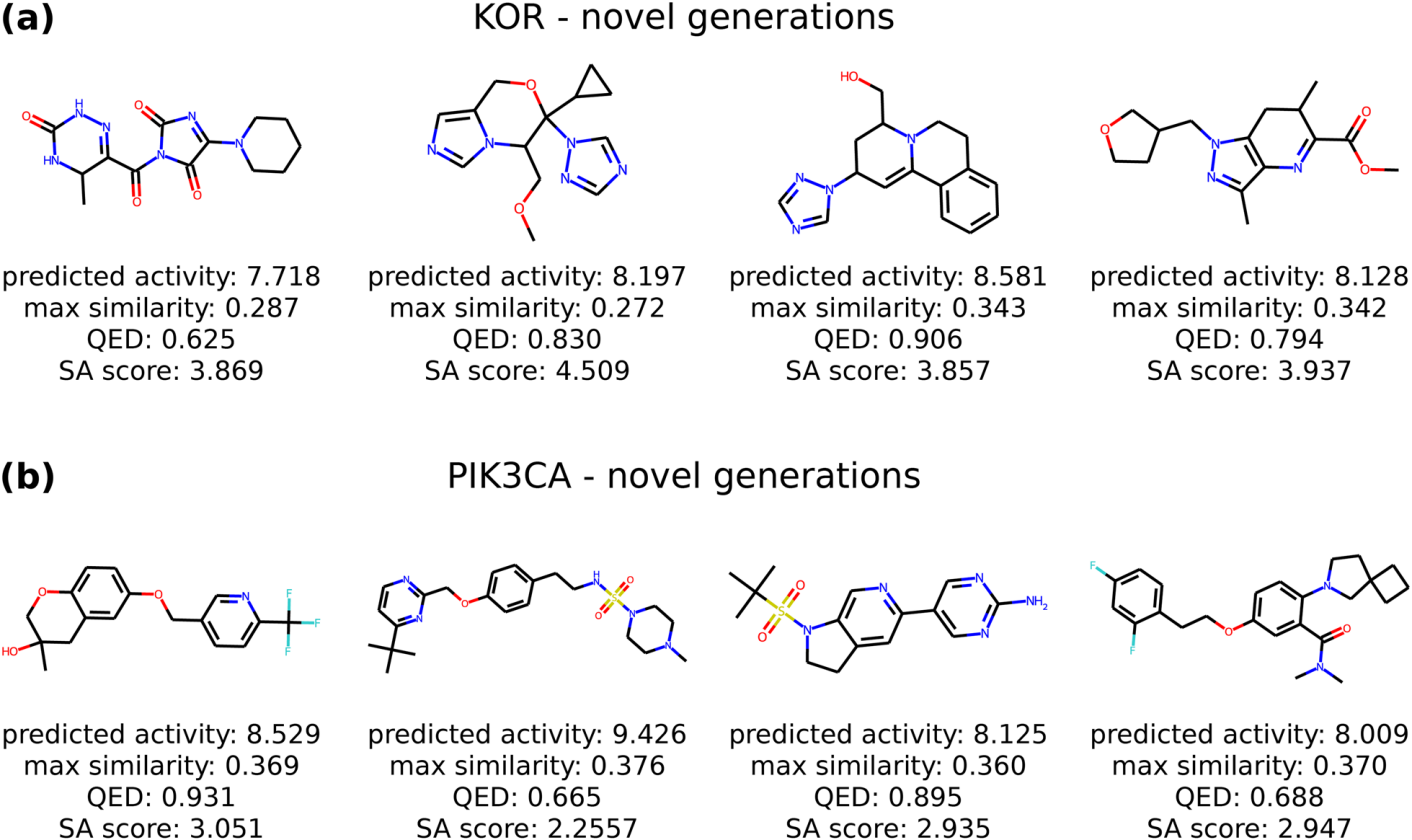
Generated novel molecules. Generated novel molecules in the 2-D structures from LOGICS. **a** novel generations from the KOR experiment. **b** novel generations from the PIK3CA experiment. The generations are selected on conditions: (1) having a Tanimoto similarity less than 0.4 to the nearest neighbor in the data and (2) predicted activity is above the activity threshold defined for each experiment

Figure 7 depicts novel structures generated from the fine-tuned LOGICS that exhibit high predicted activity. Here, we considered a generation as a novel structure when the Tanimoto similarity to its nearest neighbor in the dataset, union of ChEMBL and bioassay data, is lower than 0.4, which indicates LOGICS generated novel structures with high activity that are distinct from the molecules in the datasets used in the experiments.

## Example of binding poses of generated compounds

Computational chemistry modeling could provide structural insights by assessing the predicted binding poses of the generated optimized compounds. We investigated generated compounds that retain the same scaffold or high similarity with the reference ligands of KOR structure (PDB ID: 4DJH) and PIK3CA structure (PDB ID: 8EXL), and compared the binding poses of the generated compounds and the reference ligands.

The stability and strength of molecular interactions, crucial to ligand-receptor binding, are influenced by factors such as hydrogen bonding and the surrounding amino acid environment. In this regard, we focused on identifying and characterizing hydrogen bond formations, as they play a pivotal role in understanding ligand–protein interactions. To facilitate this analysis, we employed PyMOL [39], a widely used visualization software. The description of the process of predicting binding poses is detailed in of Additional file 1: Section 15. The hydrogen bond distance cutoff was set to 3.6 Å, which is the default setting in PyMOL and is based on the criteria from the DSSP plugin [40].

**Fig. 8:**
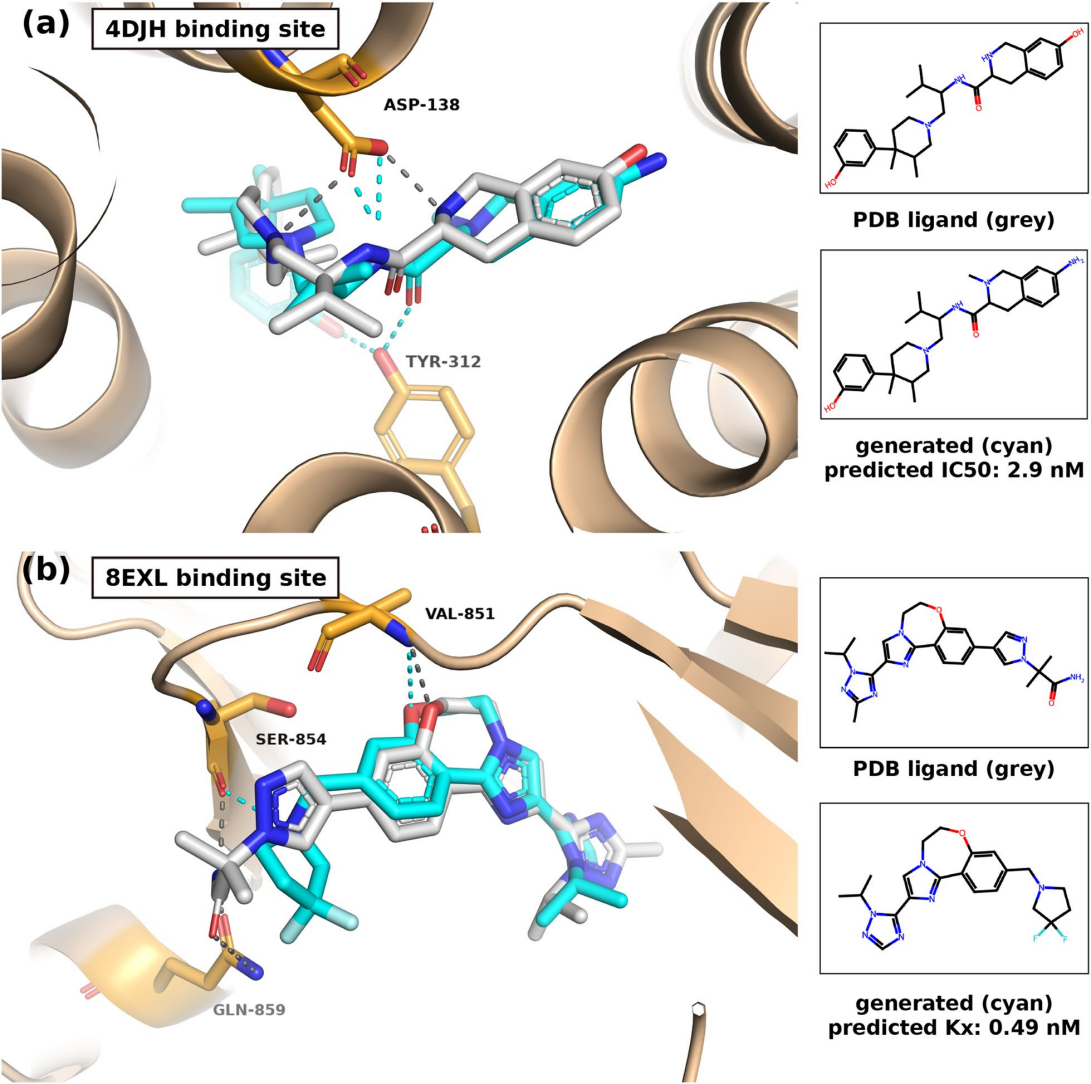
Binding site visualization with binding poses of PDB ligands and corresponding generated molecules. Using PyMol, the binding sites of (**a**) KOR protein structure (PDB ID: 4DJH) and (**b**) PIK3CA protein structure (PDB ID: 8EXL) are visualized. The ligands from each of the PDB entries are colored in grey. The generated molecules from LOGICS (cyan) having the similar scaffold as the PDB ligands are visualized. Molecular interactions between the compounds and the protein residues are shown in dotted lines, and the interacting residues' positions are specified

The binding poses of the generated compounds were examined in comparison with the original PDB ligand, which either share the same scaffold or exhibited a high structural similarity to each ligand (Fig. 8). Figure 8A illustrates the binding pose of the reference compound (PDB Ligand ID: JDC) within the KOR structure (PDB ID: 4DJH), alongside the binding pose of the generated compound. These compounds possess a shared scaffold, resulting in a noticeable overlap in their binding poses. The reference compound forms two hydrogen bonds with ASP 138, while the generated compound forms two hydrogen bonds with ASP 138 and additional two hydrogen bonds with TYR 312. Notably, the predicted IC50 of 2.9 nM indicates high activity.

In Fig. 8B, we present the binding pose of the reference compound (PDB Ligand ID: 799) within the PIK3CA structure (PDB ID: 8EXL), along with the binding pose of the generated compound. The generated compound exhibits a scaffold similarity of 0.63 (Tanimoto similarity) with the reference compound, highlighting a considerable structural resemblance. The binding poses of these compounds also exhibit overlapping characteristics. The reference compound engages in hydrogen bonds with three residues: VAL 851, SER 854, and GLN 859. Meanwhile, the generated compound forms hydrogen bonds with two residues, VAL 851 and SER 854. Also, the predicted Kx of 0.49 nM signifies high activity.

## Conclusion

In this study, we developed a novel molecular generative framework, LOGICS, which generates small molecules with the closest distribution to the target chemical space. LOGICS uses experience memory and three stages of tournament selection for objective optimization, exploration, and regularization to avoid the potential pitfalls of GPC, such as mode collapse. Bioactivity optimization experiments were conducted for two protein targets, KOR and PIK3CA, to evaluate the standard metrics and optimization metrics. The results showed that LOGICS performed better than the other methods regarding FCD and OTD, indicating that the generative distribution of the proposed model is more similar to the test set actives than the others. Several additional GPC methods showed worse performance than the pre-trained generator in terms of FCD and OTD, which indicates the potential failures of GPC. The t-SNE visualization of the learned chemical space supported the FCD and OTD scores. In addition, an increase in PredAct and PwSim did not always correspond to a closer distribution to the targeted space, further emphasizing the need to use distributional metrics. Ablation studies showed that experience memory was the most impactful component of the framework. In addition, disabling the exploration stage of tournaments resulted in a lack of generational diversity, and the regularization stage was required to generate high-quality molecules.

In summary, the LOGICS framework generates de novo chemical compounds with optimized properties. By applying experience memory and the three sophisticated stages of tournament selection for the optimization of an objective, LOGICS resolves the explore-exploit dilemma of reinforcement learning. We expect LOGICS to provide high-quality de novo chemical structure libraries with the desired properties and to contribute to the structure modification step in hit-to-lead optimization.

Though we have focused on the application of LSTM generative models, recently, many studies in molecular generative modeling demonstrated the effectiveness of using transformer-based language modeling in chemical representations. For instance, Taiga [41] is a transformer decoder-only architecture to learn the SMILES language, and policy gradient RL is performed to generate molecules with desired properties. They demonstrated that the attention mechanism of transformers improves validity and drug-likeness properties of molecular generation. Sc2Mol [42] used a transformer encoder-decoder translation model to decorate a scaffold to a desired molecule in SMILES representation. On the other hand, an application of the diffusion model has recently been studied by Levy et al. [43], where they demonstrated the capability of the denoising diffusion model for fragment-based molecular graph generation. The diffusion allowed the generation of realistic and complicated drug-like molecules. We note LOGICS to be a flexible framework in that its generative component, language LSTM in this study, can be replaced by any other molecular generative architectures such as transformers or diffusion models. We expect follow-up comparative studies using the recently developed models for the generative component of LOGICS in future.

### Supplementary Information

**Additional file 1: Figure S1.** Bioactivity label density plot of the KOR (orange) and PIK3CA (navy blue) bioassay datasets. **Figure S2.** PIK3CA bioactivity optimization performance comparison of GPC methods using three different sets of test set actives formed by each activity cutoff (> 6.0, > 8.0, > 10.0). (a) FCD and (b) OTD are calculated with each test set actives described in Table S1. **Figure S3.** Example of FCD and OTD between synthetic 2-D data points of target and generations. Two modes are assumed at (1,1) and (11,11) for the target distribution (blue). The points of generation1 (orange) were formed by adding (1,-1) to the target points. The points of generation2 (green) were sampled from the Gaussian distribution with the mean and covariance calculated by the target points. The 2-D Euclidean distance was used for OTD calculation. **Figure S4.** Predictor regression performance on KOR and PIK3CA test sets. The x-axis represents the pIC_50_ or pKx value of the test set molecule, and the y-axis represents the predicted activity by the predictor. The red dotted line is the regression line of true-to-predicted values. **Figure S5.** General overview of generator-predictor collaboration (GPC). **Figure S6.** Performance plot of GPC models during the fine-tuning phase of the KOR activity optimization case. PwSim, FCD, and OTD were calculated with the test set actives. The x-axis corresponds to the number of iterations in the fine-tuning. The vertical dotted line is the best-stopping epoch under the conditions: (1) PredAct > 7.0, (2) minimum FCD × OTD on the validation set actives. **Figure S7.** Performance plot of GPC models during the fine-tuning phase of the PIK3CA activity optimization case. PwSim, FCD, and OTD were calculated with the test set actives. The x-axis corresponds to the number of iterations used in the fine-tuning. The vertical dotted line represents the best-stopping epoch under the conditions: (1) PredAct > 8.0, (2) minimum FCD × OTD on the validation set actives. **Figure S8.** Density plots of docking scores (kcal/mol) on KOR (PDB ID: 4DJH) and PIK3CA (PDB ID: 8EXL) with generated compounds. **a**, **b** Docking score distribution of 4,000 generations from the prior generator (orange) and LOGICS fine-tuned (blue) for KOR and PIK3CA, respectively. Two-tailed *t*-test between the two distributions is performed to evaluate the p-values. **Figure S9.** Retrosynthetic prediction of the generated molecules shown in Fig. 6. The synthetic routes for (**a**), (**b**) generated molecules optimized for KOR activity, (**c**), (**d**). **Table S1.** The number of test set actives depending on the activity cutoff in the PIK3CA experiment. **Table S2.** Predictor model performance on test and validation sets for each protein target. **Table S3.** Reward function hyperparameters for policy gradient methods. **Table S4.** Tests of statistical significance in FCD and OTD metrics between LOGICS and the second-best models in Table 2. **Table S5.** Predictor performance with additional KOR datasets of varying reduced sizes and restricted structural diversity. **Table S6.** Performance of the LOGICS framework with the additional KOR datasets with reduced sizes and scaffold split. **Table S7.** Performance of REINVENT model for different σ and β reward function parameter pairs. **Table S8.** Performance comparison from the ablation study for the proposed LOGICS framework on the KOR and PIK3CA bioactivity optimization. **Table S9.** Performance comparison of the original ReLeaSE and ReLeaSE + on KOR bioactivity optimization.

## Data Availability

The source code is available at GitHub repository (https://github.com/GIST-CSBL/LOGICS).

## Abbreviations

SMILES: Simplified molecular-input line-entry system
LSTM: Long short-term memory
GPC: Generator-predictor collaboration
RNN: Recurrent neural network
FCD: Fréchet ChemNet distance
OTD: Optimal transport distance
t-SNE: T-distributed stochastic neighbor embedding
VGPC: Vanilla generator-predictor collaboration
AHC: Augmented Hill-Climb
AugMem: Augmented Memory
PredAct: Predicted bioactivity
PwSim: Pairwise similarity
QED: Quantitative estimate of drug-likeness
SA: Synthetic accessibility
