# Supplementary material for: LOGICS: Learning optimal generative distribution for designing de novo chemical structures
Source: J Cheminform. 2023 Sep 7;15:77. doi: 10.1186/s13321-023-00747-3 (PMC10483765; doi:10.1186/s13321-023-00747-3)
Supplement: Supplementary file 1 — Additional file 1: Figure S1. Bioactivity label density plot of the KOR (orange) and PIK3CA (navy blue) bioassay datasets. Figure S2. PIK3CA bioactivity optimization performance comparison of GPC methods using three different sets of test set actives formed by each activity cutoff (> 6.0, > 8.0, > 10.0). (a) FCD and (b) OTD are calculated with each test set actives described in Table S1. Figure S3. Example of FCD and OTD between synthetic 2-D data points of target and generations. Two modes are assumed at (1,1) and (11,11) for the target distribution (blue). The points of generation1 (orange) were formed by adding (1,-1) to the target points. The points of generation2 (green) were sampled from the Gaussian distribution with the mean and covariance calculated by the target points. The 2-D Euclidean distance was used for OTD calculation. Figure S4. Predictor regression performance on KOR and PIK3CA test sets. The x-axis represents the pIC50 or pKx value of the test set molecule, and the y-axis represents the predicted activity by the predictor. The red dotted line is the regression line of true-to-predicted values. Figure S5. General overview of generator-predictor collaboration (GPC). Figure S6. Performance plot of GPC models during the fine-tuning phase of the KOR activity optimization case. PwSim, FCD, and OTD were calculated with the test set actives. The x-axis corresponds to the number of iterations in the fine-tuning. The vertical dotted line is the best-stopping epoch under the conditions: (1) PredAct > 7.0, (2) minimum FCD × OTD on the validation set actives. Figure S7. Performance plot of GPC models during the fine-tuning phase of the PIK3CA activity optimization case. PwSim, FCD, and OTD were calculated with the test set actives. The x-axis corresponds to the number of iterations used in the fine-tuning. The vertical dotted line represents the best-stopping epoch under the conditions: (1) PredAct > 8.0, (2) minimum FCD × OTD on the validation set actives. F [file 13321_2023_747_MOESM1_ESM.docx]

Supplementary Information

**LOGICS: Learning optimal generative distribution for designing *de novo* chemical structures**

Bongsung Bae^1^, Haelee Bae^2^, Hojung Nam^1,2,3,§^

^1^School of Electrical Engineering and Computer Science, Gwangju Institute of Science and Technology (GIST), Buk-gu, Gwangju, 61005, Republic of Korea

^2^AI Graduate School, Gwangju Institute of Science and Technology (GIST), Buk-gu, Gwangju, 61005, Republic of Korea

^3^Center for AI-Applied High Efficiency Drug Discovery (AHEDD), Gwangju Institute of Science and Technology (GIST), Buk-gu, Gwangju, 61005, Republic of Korea

Tel. +82-62-715-2287

Fax. +82-62-715-2204

§Corresponding author

E-mail addresses:

BB: [bsbae402@gist.ac.kr](mailto:bsbae402@gist.ac.kr)

HB: haeleeeeleah@gist.ac.kr

HN: hjnam@gist.ac.kr

# Supplementary information

Supplementary description of the Introduction, Materials and Methods, and additional Results with figures and tables.

## Section 1) Supplementary description of the related studies

The studies of generative *de novo* approaches can be divided into two broad classes based on the purpose of molecular generation. The first class of research focuses on expanding the current chemical libraries. These studies use generative modeling to explore the chemical space required to retrieve novel compounds. Arús-Pous et al. [1] applied a generative recurrent neural network (RNN) for language modeling of the simplified molecular-input line-entry system (SMILES) representation to explore the unseen chemical space of 975 million molecules from the GDB-13 database. Using only 0.1% of the database as the training set, the generative model was able to discover 68.9% of the entire database. Furthermore, Arús-Pous et al. [2] demonstrated that using randomized SMILES representation as a data augmentation technique could improve the exploration ability of the generative RNN with a limited available data size. Skinnider et al. [3] performed a series of systematic benchmarks to evaluate the RNN language model with varying sizes of training datasets to determine the robustness of the model.

The second class of generative *de novo* studies focuses on property-optimized molecular generation for specific target objectives, such as increased bioactivity towards a protein target. We can further divide the studies into four categories based on how the approach retrieves the optimized molecules: (1) using evolutionary algorithms (EAs) [4-7], (2) modifying the seed molecule [8-10], (3) using generative autoencoders [11-16], and (4) inducing a higher probability of sampling molecules with desired properties [17-27].

Evolutionary algorithms (EAs) attempt to evolve a population of molecules towards high-scoring regions through genetic operations. They were previously popular tools for objective-driven molecular searches, even before the introduction of deep generative modeling [4]. Unlike deep generative models, EAs do not require training data; instead, they can only work with the given scoring function. Recent applications of EAs include ChemGE [5], GB-GA [6], EvoMol [7], and LEADD [28].

Another category of studies introduced algorithms for modifying the existing seed molecules. Some of these studies formulated the lead optimization problem as a Markov decision process in a reinforcement learning (RL) setup. A seed molecule and an objective function define an environment for reinforcement learning, where the agent learns the values of the actions, such as, adding atoms or attaching a fragment. In MolDQN, introduced by Zhou et al. [8], the modification actions are defined as valid atom addition, bond addition, and bond removal of the current molecule. They adopted the Deep Q Network learning (DQN) [29] algorithm for finding the best modification path of the seed molecule with maximal Q values. MERMAID by Erikawa et al. [30] is a modification tree learning algorithm that uses the MCTS and RNN language models trained with partial SMILES strings.

Some studies have used generative autoencoders. The studies in this category attempt to learn a robust latent space for embedding the chemical information and sampling the new molecules from the latent distribution. Gómez-Bombarelli et al. [11] introduced a variational autoencoder (VAE) working on SMILES strings and performed Bayesian optimization with a Gaussian process on the learned latent space to approximate the sampling distribution of the optimized molecules. Similarly, Winter et al. [15] used VAE to learn the continuous chemical representation of molecules and performed particle swarm optimization in a continuous space to search for a population of vectors with higher objective scores. Generative autoencoders can directly embed molecular properties as input vectors during the decoding procedure for optimized generation. Hong et al. [16] proposed the application of an adversarially regularized autoencoder for learning the molecular latent space and modified the architecture to include condition vectors for the decoder inputs. Lastly, the fourth category has been introduced in the **Introduction** section of the main text.

## Section 2) SMILES tokens used for the language model vocabulary

The language model vocabulary includes 34 SMILES tokens and 3 special tokens as follows:

H, C, N, O, F, P, S, Cl, Br, I, c, n, o, p, s, 0, 1, 2, 3, 4, 5, 6, 7, 8, 9, (, ), [, ], -, =, +, #, %, <PAD>, <BEG>, <EOS>

The special token <PAD> is used for padding a sequence, <BEG> is used for the start of a sequence, and <EOS> is used for the end of a sequence.

## Section 3) Data description

We aimed to develop a versatile framework suitable for a wide range of in-silico drug design applications. Both functional activities and binding affinity play important roles in drug discovery. We gathered pre-processed functional assay data featuring pIC_50_ values for KOR, and utilized Kd and Ki values to represent the biological activity of compounds for the PIK3CA protein. This approach allows our framework to accommodate various assay data types. We found the PIK3CA provides the largest dataset for the Kd and Ki values. Thus, we decided to test the framework on PIK3CA binding affinity optimization since the larger dataset ensures more reliable predictor performance. Similar to some studies on protein-ligand binding affinity prediction [31, 32], we took the negative natural logarithm forms of Kd and Ki values, pKd and pKi, where both become a single value, pKx, to be predicted by the regression model.

For the target proteins KOR and PIK3CA, we used different activity label thresholds to determine whether a molecule in the bioassay was active or inactive. **Figure S1** shows the distribution of activity values from each bioassay. We tried to balance the ratio of the active and inactive data counts in each experiment; thus, the threshold for KOR and PIK3CA activity was set to 7.0 and 8.0, respectively.

**
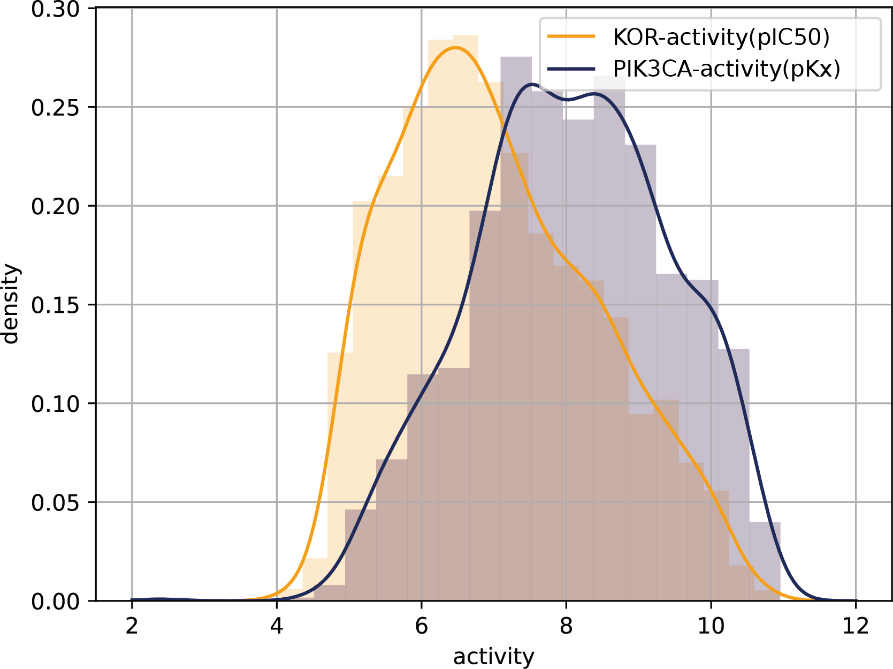
**

**Figure S1**. Bioactivity label density plot of the KOR (orange) and PIK3CA (navy blue) bioassay datasets.

## Section 4) Investigation on generative model performances in evaluations using different activity cutoffs for bioassay datasets

Here, we investigated the impact of using different activity cutoffs for bioassay datasets in the evaluation of generative models in this study. Firstly, we would like to point out that the choice of the activity cutoff doesn't affect the training or evaluation of bioactivity predictors since we adopted regression models for the prediction, which doesn't require the data label to be binary.

As mentioned in **Section 3** of **Additional File 1**, we set the activity cutoff of both pKd and pKi to be 8.0 in the PIK3CA experiment. This criterion was chosen to ensure an approximate balance between the numbers of inactive and active molecules, resulting in a "test set actives" collection that contains unseen molecules with activity levels surpassing the median of the bioassays. The "test set actives" are used to evaluate whether the fine-tuned generator's distribution is close to the target-active molecules. We suggest the balanced cutoff because if the cutoff is too low (less stringent), all the fine-tuned generators would easily find the active regions, which would diminish the significance of performance differences between the models. Whereas if the cutoff is too high (too stringent), the distributional metrics such as FCD and OTD cannot be appropriately calculated as too few "test set actives" will be used. Therefore, a balanced cutoff is crucial for ensuring a fair comparison of model performance and accurate computation of distributional metrics.

While we recommend the balanced cutoff, we have additionally investigated how the different activity cutoffs affect the evaluation of generative models. We first counted the number of test set actives on the different PIK3CA activity cutoff setups: (1) > 6.0 (less stringent), (2) > 8.0 (balanced), and (3) > 10.0 (too stringent). The counts are described in **Table S1**. If the cutoff is set to > 6.0, about 90% of the test set is labeled as test set actives. If the cutoff is set to > 10.0, only the 27 molecules (about 10%) are used to form the target active distribution for evaluating FCD and OTD.

**Table S1**. The number of test set actives depending on the activity cutoff in the PIK3CA experiment.

| PIK3CA activity cutoff | > 6.0 | > 8.0 | > 10.0 |
| --- | --- | --- | --- |
| test set actives / inactives | 221 / 26 | 115 / 132 | 27 / 220 |

We then performed the evaluation following the same process described in the Results and Discussion section, with the different activity cutoffs. The results are reported in **Figure S2**. According to **Figure S2A**, differences in FCD values of the GPC models in the less stringent cutoff (> 6.0) have been decreased compared to the balanced cutoff (> 8.0). This indicates that all the generative models have produced generative distributions that are equally close to the target actives. Consequently, it becomes challenging to discriminate between the performance of the different models. With a more stringent cutoff (>10.0), the FCD values of most of the models have increased in **Figure S2A**, while LOGICS still achieved the best FCD with a value around 43. With the cutoff (>10.0), we could clearly notice which model performed better than others, however, it could also be observed that all models failed to learn a distribution close to the target, as they all displayed an FCD value above 40. In **Figure S2B**, LOGICS and REINVENT have shown significantly better OTD performance compared to the others across all the cutoffs. Interestingly, the OTD value of LOGICS tends to decrease with a more stringent cutoff, indicating that the model has learned the close distribution to the targets with very high activity. Through the investigation, we confirmed that the bioactivity optimization performance of the LOGICS framework is not biased towards a specific threshold value for defining target active molecules.


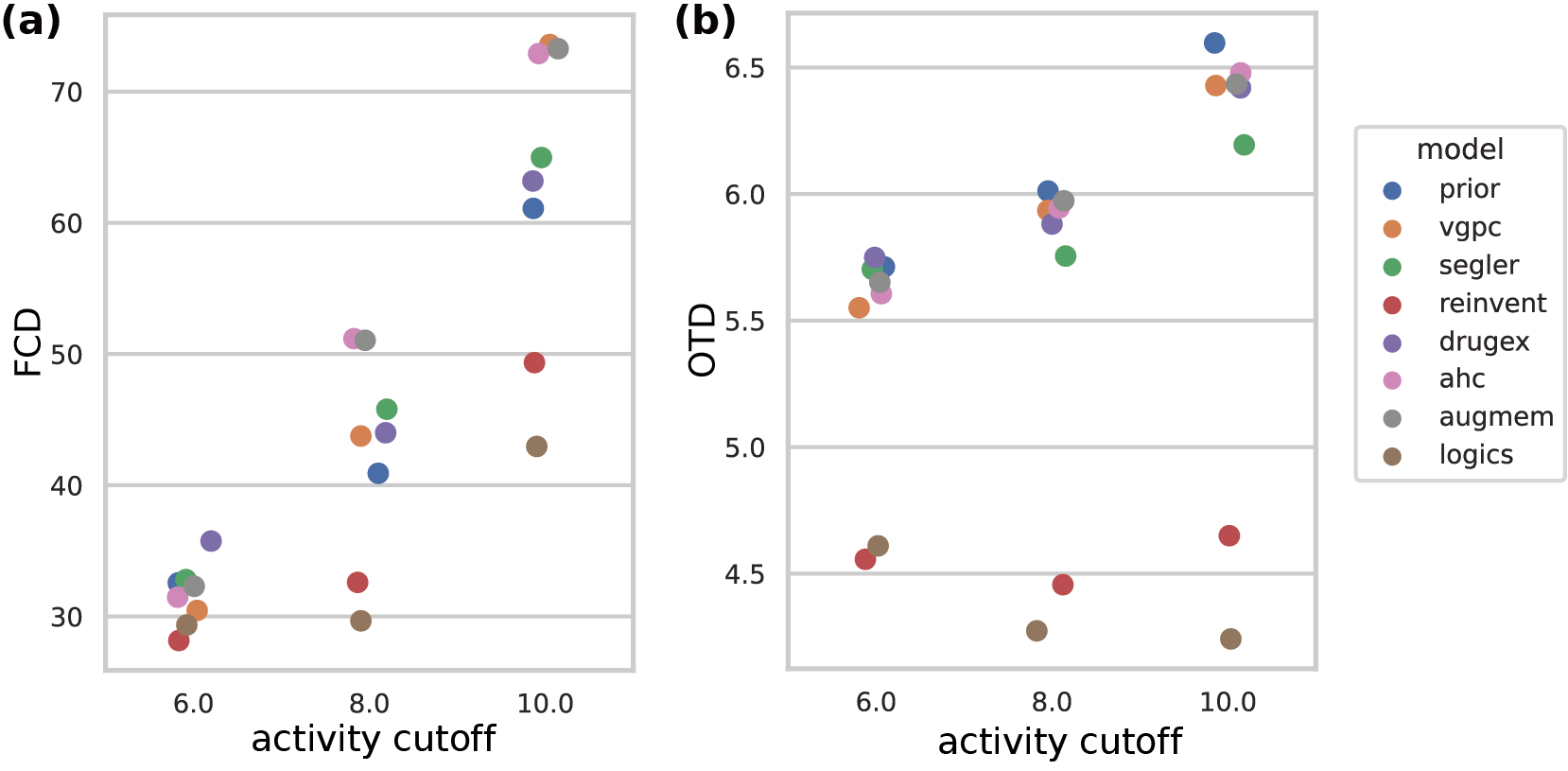
**Figure S2**. PIK3CA bioactivity optimization performance comparison of GPC methods using three different sets of test set actives formed by each activity cutoff (> 6.0, >8.0, >10.0). (a) FCD and (b) OTD are calculated with each test set actives described in **Table S1**.

## Section 5) Supplementary description of LOGICS framework

### 5-1) Bioactivity predictor

Unlike other studies that used classification models for the target activity predictor [17, 22, 33, 34], regression models for pKx and pIC_50_ value prediction were constructed in this study. By utilizing a regression model, the reward signals from weakly active molecular structures and strongly active structures can be differentiated by a smooth scoring function. For example, a bioassay record of pIC_50_ > 6.0 or 7.0 is often considered to imply active interaction of the molecule with the protein target, and classification models are trained to predict to output 1.0 value for the active interaction in such cases [22, 34]. Usually, the classifiers return an output ranging from 0.5 to 1.0, indicating confidence in its prediction, but the output is not directly translated to the exact bioactivity value. In contrast, the pIC_50_ regression model can directly predict the exact bioactivity.

The binding affinity prediction models were trained with the pre-processed bioassay datasets described in **Datasets** section of the main text. For the feature vectors of the regression models, RDKit's Morgan fingerprint using 2048 bits and a radius of 2 was used. Random forest regressors (RFR) were trained using five-fold cross-validation. Additionally, default parameters from Scikit-learn [35] (version 0.23.1) were used. Among the five models from the cross-validations, we selected the RFR model that showed the best R2 performance on the validation set. The test set performance of the selected predictor is reported in **Section 7**.

### 5-2) Generator pre-training

Generative LSTM architecture was used for language modeling of SMILES sequences. The LSTM unit obtains a SMILES token as an input, and with the input and current cell states, it outputs a multinomial probability of selecting the token for the next position. The specific architecture of the generator consists of an embedding layer for the SMILES token, a three-layer LSTM unit with a hidden state size of 512, and a final linear layer with a softmax output. The teacher forcing method was adopted to train the sequence model [2]. The first token was always set to "<BEG>", and we added "<EOS>" token at the end of every SMILES sequence in the training data. The model was constructed using PyTorch [36].

To pre-train the generator, the ChEMBL dataset from GuacaMol [37]. The batch size was set to 128, and the learning rate was initially set to 0.001. For training process stability [2], we used a custom learning rate decay strategy, in which the learning rate is multiplied by 0.8 for the decay if the average NLL in the current epoch is not smaller than the average NLL in the previous epoch minus a pre-defined constant value. We refer to the pre-trained generator as prior generator (*G_θ_*). The training for *G_θ_* is performed until it first reaches a generation validity of 95%.

## Section 6) Supplementary description of distributional metrics (FCD and OTD)

In previous studies [37, 38], FCD was used to evaluate the quality and diversity of generations by measuring the distance between the distribution of real compounds in a generic chemical database (e.g., ZINC or ChEMBL) and the distribution of generated molecules. FCD [39] was originally designed to apply Fréchet inception distance (FID), which measures the quality of generated images [40], to the chemical domain. Some recent studies in the image domain applied FID to use class-specific data to evaluate generation of images in a specific category [41]. Similarly, we applies FCD to measure the distance between the distribution of test set actives and the distribution of model generations. FCD was calculated as follows:

$$FCD=||\mu_{V}-\mu_{T}{||}^{2}+Tr(C_{V}+C_{T}-{2(C_{V}C_{T})}^{1/2})$$

where *V* is the valid generations, *T* is the test set actives, $\mu_{V}$ and $\mu_{T}$ are the means of the feature vectors generated by the ChemNet model from a previous study [39], while $C_{V}$ and $C_{T}$ are the covariances of the vectors.

FCD, as originally proposed by FID [40], assumes normal distributions on both *V* and *T*. In other words, using FCD when the target actives form a distribution with multiple modes is not desired. For example, the actual target distribution of *T* can be a mixture of Gaussians in the feature space, where the means of separate Gaussians are far apart. In this case, assuming a single Gaussian in *T* would produce a misleading FCD in that it gives a closer distance to *V* with points that loosely cover the mixture distribution. An example of this situation is illustrated in the following subsection. To avoid this pitfall, we proposed to apply another distributional metric that doesn't assume any specific forms of distribution for generation and data.

In the theory of optimal transport, the distance between two probability distributions can be measured by determining the transport plan between the two sets of masses, thus minimizing the cost of the total transportation [42]. In this study, we are only interested in discrete transport problems, where the probability distributions are represented by sets of sample points or sample molecules in our case [43]. In discrete optimal transport, the total transportation cost from point set *A* to point set *B* is calculated as follows:

$\sum_{x_{i}\in A, y_{j}\in B} T_{ij} dist(x_{i},y_{j})$ s.t. $T_{ij}\geq0,\sum_{y_{j}\in B} T_{ij}=a_{i},\sum_{x_{i}\in A} T_{ij}=b_{j}$

where $T_{ij}$ is the transport mass from point $x_{i}$ to the point $y_{j}$. Additionally, $a_{i}$ is the mass assigned to $x_{i}$ and $b_{j}$ is the mass required to be moved into $y_{j}$. The problem can be further simplified by assuming *c* = |*A*| = |*B*|, where the mass amount at every point is equal to $\frac{1}{c}$, and the transported mass is always 0 or $\frac{1}{c}$. Subsequently, the mass can only be moved from one $x_{i}$ to one $y_{j}$, and other transports from or to these points are not allowed. We can then determine the optimal transport *T* by solving the following equation:

${argmin}_{T\in R}\sum_{x_{i}\in A, y_{j}\in B} T_{ij} dist(x_{i},y_{j})$ s.t. $T_{ij}\in\{0,\frac{1}{c}\},\sum_{y_{j}\in B} T_{ij}=\frac{1}{c},\sum_{x_{i}\in A} T_{ij}=\frac{1}{c}$

where *R* is the set of all possible one-to-one mappings from *A* to *B* [44]. In this study, the OTD is the minimum total transportation cost by the linear assignment between the generated molecule *A* and the test set active *B*. This problem can be efficiently solved using a faster variant of the Hungarian method [45]. We used the implementation provided by SciPy [46]. The distance used for OTD calculation is:

$$dist(x_{i},y_{j})={10}^{1-sim(x_{i},y_{j})}-1$$

The value range of the distance is from 0 to 9, where zero means that the fingerprints of the two molecules are exactly the same and nine means that the two fingerprints have no intersection.

As stated previously, the simplified OTD metric can only be calculated when |*A*| = |*B*|. To meet this condition, we used a smaller set of generation samples whose size was equal to the number of target actives. We repeated the OTD calculation ten times with different sets of generation samples and reported the mean of the ten calculated OTDs as the OTD performance metric.

### 6-1) FCD and OTD in a synthetic 2-D example with multiple target modes

Here, we show that FCD is not an ideal metric to use when the target distribution contains multiple modes by using synthetic data points on a 2-D Euclidean space. For simplicity, we used two-dimensional Euclidean vectors instead of a hidden representation of ChemNet [39]. Euclidean distance between two vectors was used for OTD calculation. In **Figure S3**, we created a hypothetical target distribution with two distant modes and sampled 100 points (blue). Generation1 samples (orange) were formed by moving the target points in the (1,-1) direction. We calculated the mean and covariance of the target points and used them to form a Gaussian distribution and then sampled 100 points (green) for generation2. By visually inspecting **Figure S3**, generation1 can be seen to be closer to the target than generation2. A proper distribution-based metric should show a smaller distance between the target and generation1. However, when we calculated the FCD between the target and each generation, we observed that the FCD was smaller for generation2. In contrast, the OTD showed a lower distance between the target and generation1, which corresponds to the actual landscape of **Figure S3**, where the target and generation1 are closely distributed. This indicates that OTD is a more appropriate metric when the target has multiple modes.


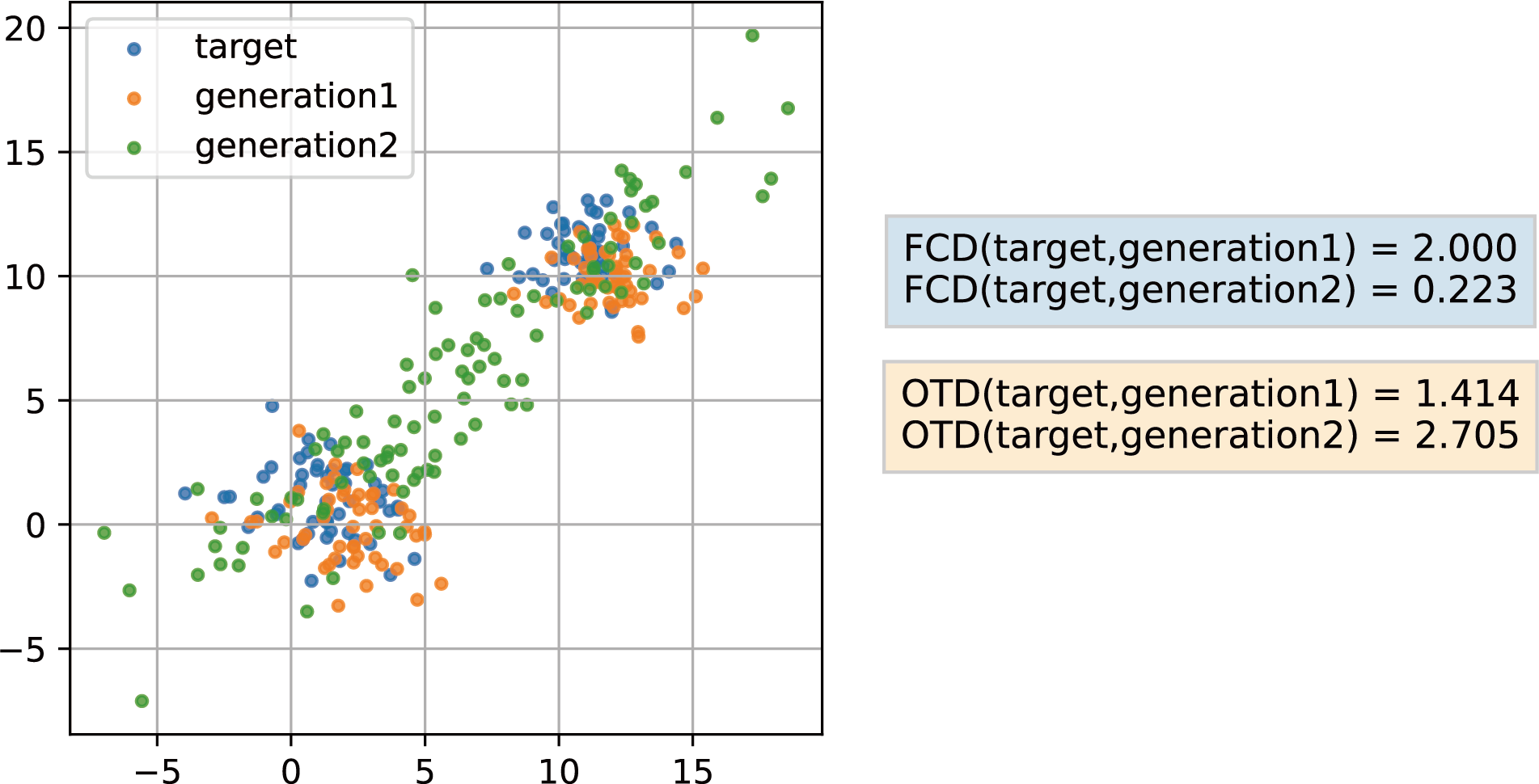


**Figure S3**. Example of FCD and OTD between synthetic 2-D data points of target and generations. Two modes are assumed at (1,1) and (11,11) for the target distribution (blue). The points of generation1 (orange) were formed by adding (1,-1) to the target points. The points of generation2 (green) were sampled from the Gaussian distribution with the mean and covariance calculated by the target points. The 2-D Euclidean distance was used for OTD calculation.

## Section 7) Bioactivity predictor performance

**Table S2**. Predictor model performance on test and validation sets for each protein target.

|  | Validation | | Test | |
| --- | --- | --- | --- | --- |
|  | MSE^a^ | R^2^ | MSE^a^ | R^2^ |
| KOR predictor | 0.4900 | 0.7570 | 0.5416 | 0.7132 |
| PIK3CA predictor | 0.3498 | 0.8080 | 0.4867 | 0.7594 |

^a^ Mean squared error


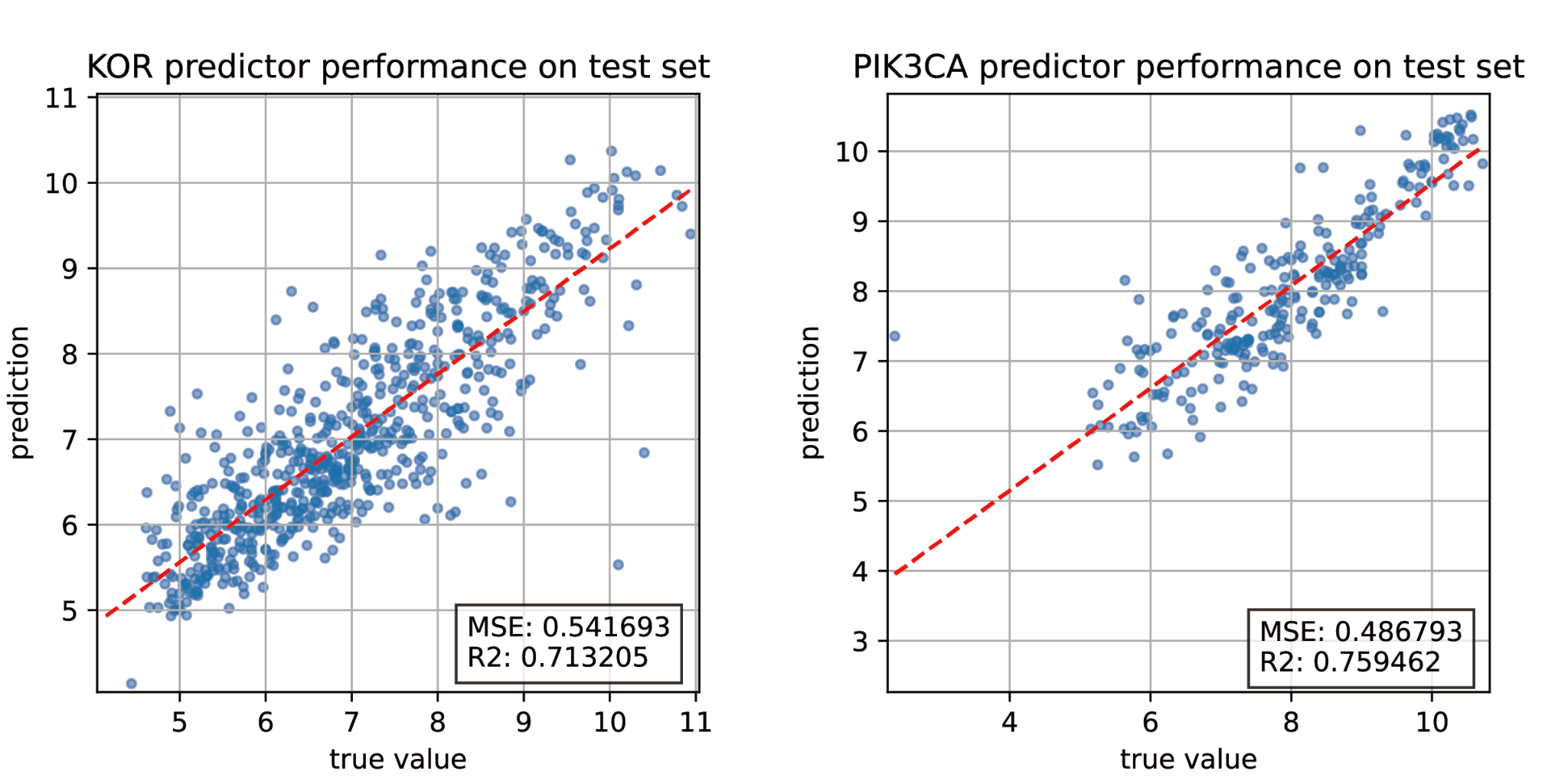


**Figure S4**. Predictor regression performance on KOR and PIK3CA test sets. The x-axis represents the pIC_50_ or pKx value of the test set molecule, and the y-axis represents the predicted activity by the predictor. The red dotted line is the regression line of true-to-predicted values.

## Section 8) Brief description of the other GPC methods used in this paper


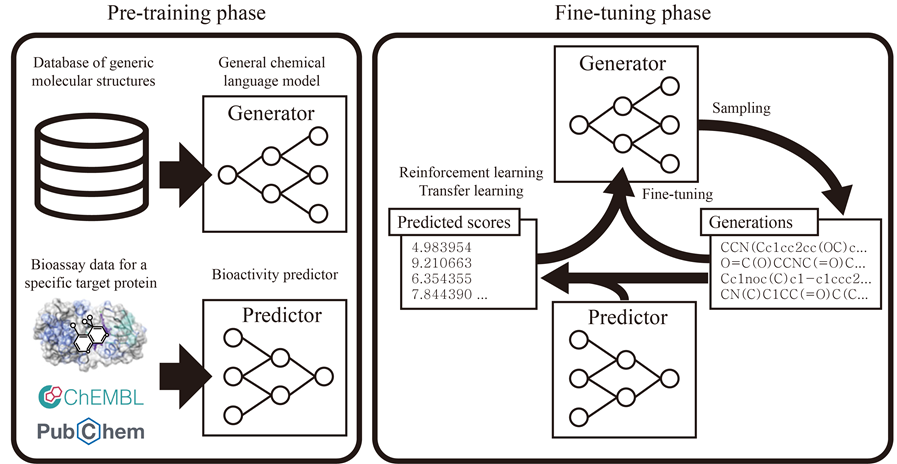


**Figure S5**. General overview of generator-predictor collaboration (GPC).

### 8-1) Methods using negative log likelihood (NLL) minimization on predicted actives

Vanilla GPC (VGPC) is the simplest type of GPC. In the fine-tuning phase, the agent samples N sequences. The invalid SMILES are then filtered out, and the sampled molecules are scored using the predictor. The top N/5 molecules are retrieved based on the scores and used to fine-tune the agent by minimizing NLL. The procedure is repeated for pre-defined epochs. N was set to 20,000 in these experiments. This method is widely known as Hillclimb-MLE [47], and adopted as a baseline in many studies of molecular generative RNNs [37, 48].

Segler [20] is a transfer learning approach in which the memory Q of the predicted actives is maintained throughout the fine-tuning phase. Before beginning fine-tuning, memory Q is initialized with the predicted actives among 100,000 generations from the prior generator. In the fine-tuning phase, the agent samples N sequences, filters out the invalid SMILES, and scores the samples. For the target activity threshold (7.0 for KOR and 8.0 for PIK3CA), samples with activity below the threshold are removed. The new predicted actives are inserted into memory Q, and the agent is fine-tuned with the molecules in Q. The procedure is repeated for the pre-defined epochs. N was set as 10,000 in these experiments.

### 8-2) Policy gradient methods and reward functions

Among the GPC methods used for the experiments in this study, REINVENT [17], DrugEx [22], ReLeaSE [19] Augmented Hill-Climb (AHC) [48], and Augmented Memory (AugMem) [49] comprise the policy gradient methods, and their learning objectives include the reward values to be maximized.

- REINVENT, AHC, and AugMem minimize the following cost function that includes the augmented likelihood:

$$J_{\varphi}(x_{1:T})={(logG_{\theta}(x_{1:T})+\sigma R(x_{1:T})-logG_{\varphi}(x_{1:T}))}^{2}$$

Augmented Hill Climb (AHC) is a recent modification to the REINVENT RL framework. Its agent fine-tuning phase is a simple hybrid strategy based on both Hill-Climb and REINVENT policy gradient approaches. For each iteration, the agent first samples *m* molecules. The rewards for the molecules are calculated with the predictor, and top-*k* rewarding molecules are selected, based on Hill-Climb. The agent is only fine-tuned with the *k* molecules, based on REINVENT loss function. They also used a diversity filter (DF) for reward calculation, where the generated molecules having the similar scaffolds to the previously generated ones from the earlier iterations receive penalized rewards.

Augmented Memory (AugMem) is another recent modification to the REINVENT framework. With emphasis on the sample efficiency of goal-directed generative models, they proposed an experience replay mechanism in conjunction with Selective Memory Purge as an extension to the REINVENT framework. In a fine-tuning phase, they maintain a replay buffer, similar to the experience memory of LOGICS. For each iteration in the fine-tuning phase, the agent first samples a batch of SMILES, and the rewards for the samples are calculated. Then, the agent is fine-tuned with the batch, and the buffer is updated with the new molecules. For the update of the buffer, the molecules in the buffer are purged based on a diversity filter (DF). If the molecule receives a penalized reward of zero by the DF, then the example is removed from the buffer. During each iteration, the agent is also updated multiple times with the replay buffer, by augmenting each SMILES as a randomized SMILES representation [2] of the same molecule.

- ReLeaSE and DrugEx minimize the following loss function from the REINFORCE policy gradient algorithm:

$$J_{\varphi}(x_{1:T})={-\sigma R(x_{1:T})logG_{\varphi}(x_{1:T})}$$

DrugEx uses an exploration strategy to generate samples where, at time step t, ε = 0.1 chance to use the prior $G_{\theta}(x_{t}|x_{1:t-1})$ probability to sample the $x_{t}$ token. ReLeaSE applies the discounted reward for a sampled sequence, where the reward for each token position $x_{t}$ is multiplied by $\gamma^{t}$. $\gamma$ was set to 0.97 for the experiments.

$R(x_{1:T})$ is the reward value of a sampled sequence and is defined as the maximization objective of the policy gradient method. $\sigma$ is the scaling constant for the rewards, and a different $\sigma$ value was set for each model. We used the following transformation from the predicted bioactivity to the reward:

$$R(x_{1:T})=tanh(0.3(f(x_{1:T})-\beta))$$

$R(x_{1:T})=-0.5$ if the given sequence $x_{1:T}$ is invalid

where $f(x_{1:T})$ is the predicted bioactivity of a given sequence $x_{1:T}$. The hyperbolic tangent is applied to transform the predicted value into the range from -1 to 1, which significantly contributes to the stabilization of the policy gradient learning. $\beta$ is an offset used to distinguish between high and low bioactivity. Different $\beta$ values are used for KOR and PIK3CA bioactivities because the distributions of activity values from the bioassay data of the two targets are different (**Figure S1**). **Table S3** lists the $\sigma$ and $\beta$ values used for each policy gradient method.

**Table S3**. Reward function hyperparameters for policy gradient methods.

|  | Protein  target | Policy gradient method | | | | |
| --- | --- | --- | --- | --- | --- | --- |
|  |  | REINVENT | DrugEx | ReLeaSE | AHC | AugMem |
| σ | KOR | 10 | 10 | 10 | 15 | 20 |
|  | PIK3CA | 14 | 10 | 10 | 15 | 20 |
| β | KOR | 6.2 | 6.2 | 6.2 | 6.2 | 6.2 |
|  | PIK3CA | 6.8 | 6.8 | 6.8 | 6.8 | 6.8 |

## Section 9) Additional information on the performance of the fine-tuned generator


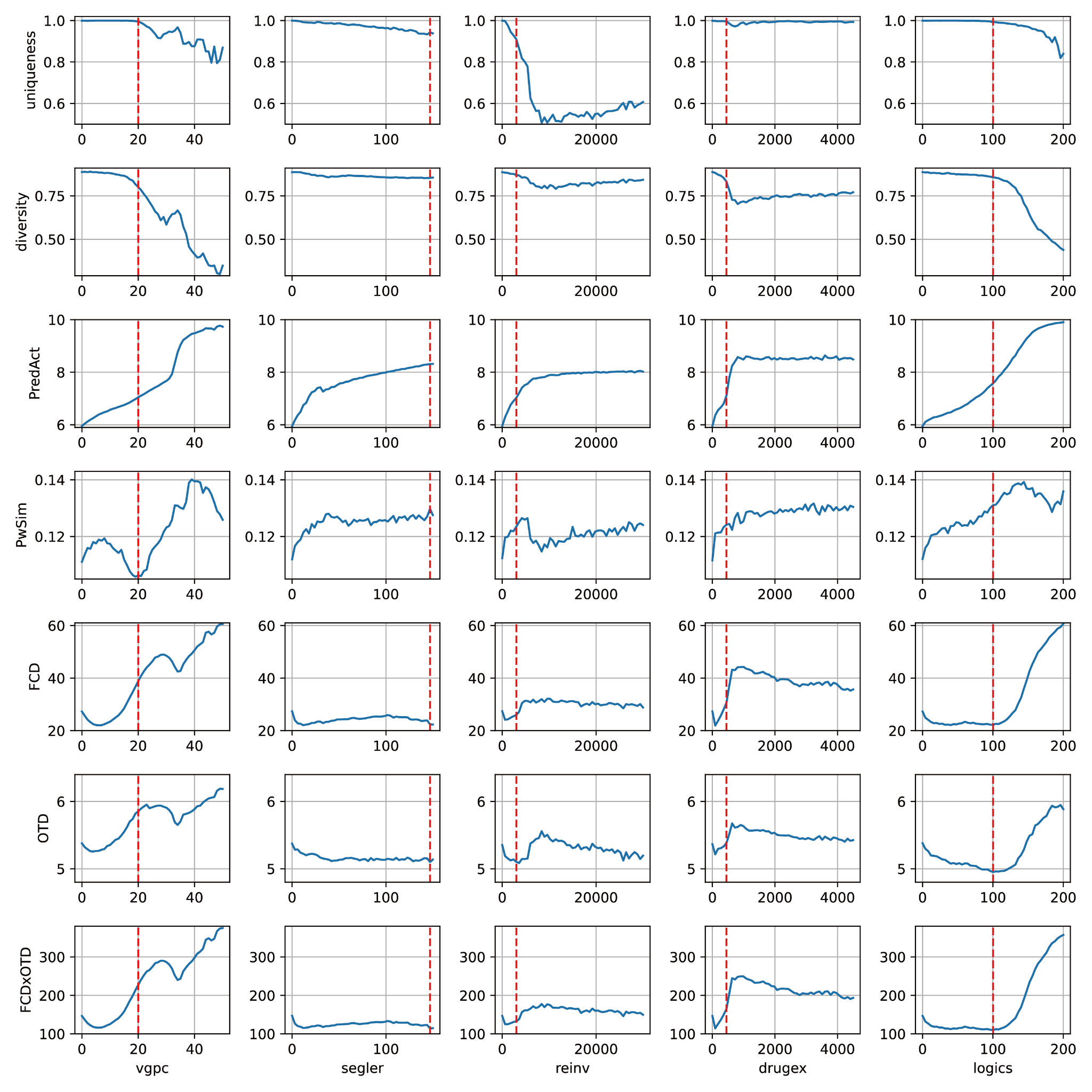


**Figure S6**. Performance plot of GPC models during the fine-tuning phase of the KOR activity optimization case. PwSim, FCD, and OTD were calculated with the test set actives. The x-axis corresponds to the number of iterations in the fine-tuning. The vertical dotted line is the best-stopping epoch under the conditions: (1) PredAct > 7.0, (2) minimum FCD×OTD on the validation set actives.


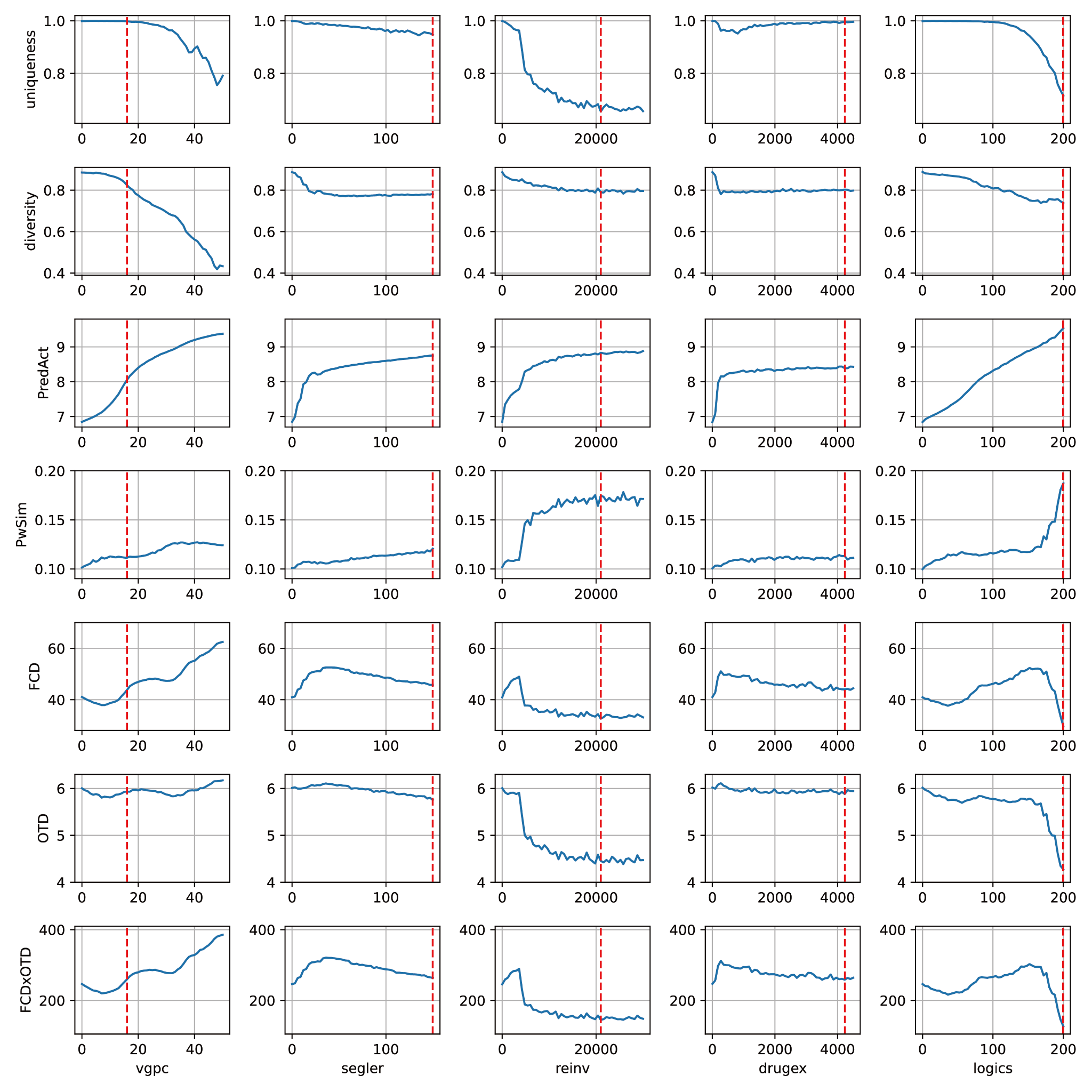


**Figure S7**. Performance plot of GPC models during the fine-tuning phase of the PIK3CA activity optimization case. PwSim, FCD, and OTD were calculated with the test set actives. The x-axis corresponds to the number of iterations used in the fine-tuning. The vertical dotted line represents the best-stopping epoch under the conditions: (1) PredAct > 8.0, (2) minimum FCD×OTD on the validation set actives.

## Section 10) Tests of statistical significance in FCD and OTD performance of LOGICS

In **Table 2**, we performed evaluation three times for each model with three different sets of 20,000 samples generated by the model, and reported the mean and standard error of the mean calculated by the three values of each performance metric. To clarify the statistical significance of LOGICS performance in OTD and FCD, we performed a *t*-test comparing LOGICS to the second-best performing model (**Table S4**). In the KOR case of **Table S4**, FCD and OTD values from LOGICS are significantly better than the second-best model Segler (two-tailed *t*-test, p-value = 1.95 x 10^-2^ for FCD, 1.32 x 10^-4^ for OTD). Also, in the PIK3CA case, FCD and OTD values from LOGICS are significantly better than the second-best model REINVENT (two-tailed *t*-test, p-value = 1.87 x 10^-5^ for FCD, 1.44 x 10^-3^ for OTD). According to the statistical tests, LOGICS is clearly capable of learning the significantly closer distribution to the test set actives than other methods.

**Table S4**. Tests of statistical significance in FCD and OTD metrics between LOGICS and the second-best models in **Table 2.**

|  | Metric | Model 1 (mean±sem^*^) | Model 2 (mean±sem^*^) | p-value |
| --- | --- | --- | --- | --- |
| KOR | FCD | LOGICS (22.2±0.01) | Segler (22.3±0.03) | 1.95 x 10^-2^ |
|  | OTD | LOGICS (4.95±0.00) | Segler (5.09±0.00) | 1.32 x 10^-4^ |
| PIK3CA | FCD | LOGICS (29.4±0.10) | REINVENT (32.7±0.08) | 1.87 x 10^-5^ |
|  | OTD | LOGICS (4.27±0.02) | REINVENT (4.47±0.02) | 1.44 x 10^-3^ |

^*^ standard error of the mean

*t*-test is performed with three metric values, each from LOGICS (Model 1) and the second-best model (Model 2). The three metric values are calculated from the three independent sets of 20,000 samples from each model.

## Section 11) Robustness of LOGICS in data size and structural diversity

It is widely common that data size and structural diversity of biological assays could vary dramatically depending on the type of protein target. Here, we investigated the robustness of LOGICS in the case of artificially reduced data size and restricted structural diversity of training datasets.

Firstly, to evaluate the effect of data size on our framework, we prepared three additional KOR bioassay datasets of varying reduced sizes. These datasets, named "1/2-Data", "1/4-Data", and "1/10-Data", were generated by randomly eliminating 50%, 75%, and 90% of the CV set from the original set, respectively.

Secondly, to investigate the effect of structural diversity of data, we also developed another KOR dataset, which split the test set using the scaffold split method from DeepChem [50]. This ensured the CV set and the test set do not contain molecules sharing a common scaffold. We have trained the random forest regressor on the 5-fold CV of each dataset, and evaluated the regressors with each test set. The new datasets and the newly trained regression models' performance on the test sets are reported in **Table S5**.

**Table S5**. Predictor performance with additional KOR datasets of varying reduced sizes and restricted structural diversity.

|  | Data set size | | | Predictor performance | |
| --- | --- | --- | --- | --- | --- |
| KOR dataset | Total | 5-fold CV^a^ | Test | MSE^b^ | R^2c^ |
| Original | 3,881 | 3,230 | 651 | 0.541 | 0.713 |
| 1/2-Data | 2,266 | 1,615 | 651 | 0.688 | 0.482 |
| 1/4-Data | 1,461 | 810 | 651 | 0.702 | 0.392 |
| 1/10-Data | 976 | 325 | 651 | 0.973 | 0.067 |
| Scaf-Split | 3,881 | 3,104 | 777 | 0.794 | 0.309 |

^a^ Cross validation

^b^ Mean squared error by the regression model on the test set

^c^ R^2^ (coefficient of determination) by the regression model on the test set

As indicated in **Table S5**, diminishing the training set size evidently deteriorates the performance of the activity regression models. There is a clear pattern: as the size of the data decreases, prediction error escalates, and the correlation between predicted and true activity values worsens. Also, compared to the original dataset, the scaffold-split dataset hugely degrades the predictor by reducing the test set R^2^ value from 0.713 to 0.309.

With these degraded predictors, we fine-tuned the generators following the LOGICS framework. For the evaluation of the fine-tuned generators of reduced dataset cases, we used test set actives from the original KOR dataset. For the evaluation of the Scaf-Split case, we used the scaffold-split test set. The original predictor is used for PredAct calculation to provide more accurate predictions on KOR activity. The performance of each fine-tuned generator is reported in **Table S6**.

**Table S6**. Performance of the LOGICS framework with the additional KOR datasets with reduced sizes and scaffold split

|  |  | Original | 1/2-Data | 1/4-Data | 1/10-Data | Scaf-Split |
| --- | --- | --- | --- | --- | --- | --- |
| Standard | Validity | 0.984 | 0.984 | 0.986 | 0.994 | 0.980 |
|  | Uniqueness | 0.992 | 0.999 | 0.997 | 0.999 | 0.997 |
|  | Novelty | 0.986 | 0.991 | 0.992 | 0.998 | 0.988 |
|  | Diversity | 0.857 | 0.850 | 0.853 | 0.760 | 0.859 |
| Optimization | PredAct | 7.570 | 7.021 | 7.043 | 7.022 | 7.099 |
|  | PwSim | 0.131 | 0.127 | 0.130 | 0.123 | 0.130 |
|  | FCD | 22.205 | 25.129 | 24.216 | 42.414 | 22.483 |
|  | OTD | 4.952 | 5.096 | 5.000 | 5.481 | 5.044 |

The fine-tuning process was performed with the predictor trained on each data provided in **Table S5**. PredAct was evaluated with the original predictor to provide a more accurate prediction.

According to **Table S6**, modifications in data size and structural diversity had negligible effects on standard metrics such as validity, uniqueness, novelty, and diversity. In optimization metrics of 1/2-Data and 1/4-Data, we observed minor increases in FCD and OTD values. Despite the reduced data size, these LOGICS frameworks were still capable of learning a distribution closely resembling the test set actives. However, LOGICS with 1/10-Data exhibited a substantial decline in OTD and FCD performance compared to the others in **Table S6**. This implies that the degraded activity predictor of 1/10-Data, with a mere 0.067 R^2^ as shown in **Table S5**, could disrupt the LOGICS fine-tuning process. This is evident as the generator records 42.414 FCD and 5.481 OTD, which are inferior to the prior results from **Table 1**.

**Table S6** also shows the performance of LOGICS with the scaffold-split dataset as 22.483 FCD and 5.044 OTD which is similar to those of original LOGICS with randomly split dataset. This result indicates LOGICS was able to learn the comparable distribution close to the unseen test set actives, even when the scaffolds of the training set do not have the scaffolds of the test set. We presume the exploration ability of LOGICS framework enabled the generator to discover the unseen scaffolds during the fine-tuning process.

Based on our findings, for LOGICS to reliably fine-tune, the performance of the activity predictor should ideally attain R^2^ score of approximately 0.3 and above, as evidenced by the LOGICS implementations with modified datasets (1/2-Data, 1/4-Data, Scaf-Split) in **Table S6**. If the predictor's performance declines below R^2^ of 0.3, LOGICS may struggle to extract meaningful information about the target activity, leading to suboptimal generator performance, as exhibited in the case of 1/10-Data in **Table S6**. Additionally, LOGICS is robust with respect to the structural diversity of the training data, given its ability to navigate through a diverse chemical space to identify unseen scaffolds.

## Section 12) Practical benefit of LOGICS

Aside from the fact that LOGICS is capable of learning the closest distribution to the desired unseen molecules as shown in **Table 2**, one of the benefits of using LOGICS is that it doesn't require a reward function engineering, or parameter tuning for reward functions, unlike the most RL-based policy gradient models. In this study, we have implemented various policy gradient methods in our framework, such as DrugEx and REINVENT, and we have noticed that the way of transforming pKx or pIC50 to a reward could make a huge difference in the performance of the RL agents. As described in **Section 8** of **Additional File 1**, the predicted activity value is transformed to a reward by tanh function with an offset β, and the reward is amplified by a scaling constant σ. Finding appropriate values for σ and β is a part of hyperparameter tuning of the policy gradient methods.

Here, we report failures in the REINVENT model due to inappropriate values for σ and β in **Table S7**. In **Table S7**, we have reported some extreme cases encountered in our experiments, while performing trials and errors to find the best parameter values. For example, in the KOR case, using 10 for σ and 8.0 for β completely decimates the REINVENT agent, as diversity 0.0 indicates it only generates a single molecule repeatedly. In the PIK3CA case, the inappropriate values for (σ, β) such as (14, 9.0) and (50, 6.8) caused the REINVENT to fail to learn the close distribution to the target actives, as OTD and FCD values are too large compared to the best value pair (14, 6.8). Therefore, policy gradient methods like REINVENT are very sensitive to the settings of parameters in the reward function, thus, it is necessary to invest much time into the parameter search.

**Table S7**. Performance of REINVENT model for different σ and β reward function parameter pairs

| Protein  target | Reward parameters | | REINVENT performance | | | |
| --- | --- | --- | --- | --- | --- | --- |
|  | σ | β | Diversity | PredAct | FCD | OTD |
| KOR | 10* | 6.2* | 0.870* | 7.024* | 25.968* | 5.102* |
|  | 10 | 8.0 | 0.0 | 7.941 | 63.325 | 7.097 |
|  | 50 | 6.2 | 0.586 | 9.062 | 48.442 | 5.277 |
| PIK3CA | 14* | 6.8* | 0.789* | 8.831* | 32.597* | 4.456* |
|  | 14 | 9.0 | 0.863 | 7.517 | 48.599 | 6.013 |
|  | 50 | 6.8 | 0.737 | 8.501 | 58.305 | 6.017 |

* best σ and β values found for REINVENT. These parameters are used in the model comparison of the **Results and Discussion** section.

On the other hand, LOGICS eliminates the need for reward function engineering. Instead of relying on policy gradients influenced by rewards, the agent update in LOGICS operates based on the maximum likelihood of the selected generated SMILES. As described in the Methods section, LOGICS selects the fine-tuning candidates by tournament selection, which compares the two molecules' predicted activities. This means the selection mechanism simply runs with the ranks between the generated molecules. In other words, the LOGICS agent is properly trained when it knows which molecule is better than the other, irrelevant to the reward transformation. Therefore, LOGICS users can bypass the time-consuming task of fine-tuning reward function parameters, a step that users of policy gradient methods, such as REINVENT, must undertake.

## Section 13) Ablation study - performance tables

**Table S8**. Performance comparison from the ablation study for the proposed LOGICS framework on the KOR and PIK3CA bioactivity optimization

|  |  |  | LOGICS | No- Memory | No- Exploration | No- Regularization | Select-TopN/2 |
| --- | --- | --- | --- | --- | --- | --- | --- |
| KOR | Standard | Validity^a^ | 0.984 | 0.881 | 0.999 | 0.829 | 0.985 |
|  |  | Uniqueness^b^ | 0.992 | 1.000 | 0.068 | 1.000 | 0.999 |
|  |  | Novelty^c^ | 0.986 | 0.999 | 0.914 | 0.998 | 0.994 |
|  |  | Diversity^d^ | 0.857 | 0.848 | 0.760 | 0.880 | 0.838 |
|  | Optimization | PredAct^e^ | 7.570 | 7.032 | **9.238** | 7.003 | 7.727 |
|  |  | PwSim^f^ | 0.131 | 0.124 | **0.176** | 0.123 | 0.132 |
|  |  | FCD^g^ | 22.20 | 26.67 | 29.01 | **20.75** | 25.11 |
|  |  | OTD^h^ | 4.952 | 5.439 | **4.035** | 5.300 | 5.082 |
| PIK3CA | Standard | Validity^a^ | 0.995 | 0.941 | 0.999 | 0.890 | 0.993 |
|  |  | Uniqueness^b^ | 0.715 | 1.000 | 0.009 | 1.000 | 0.441 |
|  |  | Novelty^c^ | 0.994 | 0.999 | 0.908 | 0.998 | 0.997 |
|  |  | Diversity^d^ | 0.737 | 0.833 | 0.270 | 0.845 | 0.460 |
|  | Optimization | PredAct^e^ | 9.539 | 8.0172 | 9.788 | 8.088 | **10.34** |
|  |  | PwSim^f^ | 0.187 | 0.112 | 0.284 | 0.112 | **0.272** |
|  |  | FCD^g^ | 29.66 | 40.34 | 25.57 | 39.25 | **16.83** |
|  |  | OTD^h^ | 4.273 | 5.940 | 3.976 | 5.881 | **3.900** |

^a^ Validity is the ratio of valid generations to 20,000 generations from the model.

^b^ Uniqueness is the ratio of unique generations to the valid generations.

^c^ Novelty is the ratio of unique generations that are not found in the pre-training dataset.

^d^ Diversity measures how dissimilar the 1,000 generations are.

^e^ PredAct is the mean of predicted activities of the valid generations.

^f^ PwSim is the mean of pairwise similarities between generations and test set actives.

^g^ FCD is the Fréchet Chemnet Distance between generations and the test set actives.

^h^ OTD is the optimal transport distance between generations and the test set actives.

## Section 14) Modification of ReLeaSE to incorporate experience memory and tournament selections

We presumed that the experience memory and selection mechanisms could benefit other GPC methods that struggle to learn the optimal distribution. ReLeaSE, introduced in a previous study, was one of the early attempts to adopt the policy gradient algorithm in *de novo* design by formulating molecule generation as a sequential decision-making process [19]. ReLeaSE did not enforce any exploration strategies in its learning process, which presumably leads to mode collapse in the fine-tuning phase. We tested the ReLeaSE method in the KOR bioactivity optimization case, initialized with our pre-trained prior model. **Table S9** shows the results of fine-tuning with the original ReLeaSE algorithm, which has worse FCD and OTD values than the prior model. We added LOGICS-like experience memory and three-stage tournament selection mechanisms to the ReLeaSE fine-tuning phase and named the modified algorithm *ReLeaSE+*. Specifically, the training examples were sampled from both the memory and the agent, and only the surviving examples from the tournaments were used to apply the policy gradients. As shown in **Table S9**, ReLeaSE+ achieved overall improved performance metrics, and the generations were closer to the test set actives of KOR.

**Table S9**. Performance comparison of the original ReLeaSE and ReLeaSE+ on KOR bioactivity optimization

|  |  | Prior | ReLeaSE | ReLeaSE+^a^ |
| --- | --- | --- | --- | --- |
| Standard | Validity | 0.953 | 0.880 | 0.986 |
|  | Uniqueness | 0.999 | 0.952 | 0.997 |
|  | Novelty | 0.948 | 0.992 | 0.975 |
|  | Diversity | 0.889 | 0.844 | 0.853 |
| Optimization | PredAct | 5.951 | 7.016 | **7.053** |
|  | PwSim | 0.109 | 0.119 | **0.126** |
|  | FCD | 27.327 | 31.007 | **25.532** |
|  | OTD | 5.384 | 5.513 | **5.110** |

^a^ ReLeaSE+ modifies the ReLeaSE method by incorporating the memory and selection mechanisms in LOGICS.

## Section 15) Docking analysis details and docking score distributions

Here, we performed in-depth validation on the generated compounds of LOGICS. We have conducted further investigations into the binding potential of the generated compounds via docking studies. Detailed process of docking analysis is described as follows.

We performed molecular docking analysis with the generated compounds on KOR and PIK3CA protein targets. Regarding KOR 3D structure, since there were no registered PDB entries for KOR from Cavia porcellus, the organism used in the KOR bioassay (ID: CHEMBL3952) [27], we instead opted for a representative KOR entry from Homo sapiens (4DJH). For 3D structure of PIK3CA protein, we used 8EXL from PDB as it has the best resolution of 1.99Å among all PDB entries of PIK3CA structures without any mutation on the protein sequence and having interaction with ligands. The binding pocket used for docking in 8EXL was the well known ATP-binding site for competitive inhibitors.

We sampled 4,000 molecules each from the prior generator, the LOGICS generator fine-tuned for KOR activity (LOGICS-KOR), and the LOGICS generator fine-tuned for PIK3CA activity (LOGICS-PIK3). We performed docking between 4DJH and the generated molecules from prior and LOGICS-KOR. We then performed docking between 8EXL and the generated molecules from the prior and LOGICS-PIK3. We used QuickVina2 [51], a fast and accurate docking software based on AutoDock Vina [52]. The docking simulations were carried out with the ligand treated as flexible, while the protein molecules were maintained in a rigid state. We separated the protein-ligand complex into individual protein and ligand components. To prepare the protein for docking, we first converted its .pdb file into .pdbqt format, which involved the addition of hydrogen bonds and charges. The compound, initially represented in a SMILES 2D structure, underwent a conversion to a 3D structure using OpenBabel [53] after the addition of hydrogen atoms. The resulting compound's .pdb file was subsequently converted to .pdbqt format as well. For the docking simulation, we defined the docking site by creating a cube that encompassed the relevant area. The dimensions of this cube were set to 20Å x 20Å x 20Å to cover the binding site. Moreover, we established specific center grid coordinates, namely -17.45, 14.07, and 29.67 for PIK3CA(8EXL) along the X, Y, and Z axes, respectively, and 3.68, -24.01, and 59.47 for KOR(4DJH) along the X, Y, and Z axes, respectively. To ensure comprehensive exploration during the docking process, we set the exhaustiveness parameter to 30. Additionally, the docking calculations were performed using 15 CPU cores, and we obtained the top 10 binding modes as part of the results. For **Figure S8**, we selected the best binding score from among the 10 modes.

Also, we applied a redocking procedure to evaluate the binding energy values of known inhibitors. Redocking involves docking a known ligand back into its original receptor with the aim of reproducing the co-crystallized binding geometry and orientation of the ligand when it was bound to the macromolecule in a rigid state. The process consists of removing the ligand molecule from the receptor and then re-docking it. Through our redocking experiments, we obtained docking scores of -10.6 and RMSD of 1.785Å for the target protein PIK3CA with the known inhibitor of 8EXL (PDB Ligand ID: 799), and docking scores of -10.6 and RMSD of 2.740Å for the target protein KOR with the known inhibitor of 4DJH (PDB Ligand ID: JDC). The docking scores indicate a strong binding interaction between the ligands and their respective target proteins, suggesting that the known inhibitors have a favorable affinity for their receptors. Moreover, the RMSD values demonstrate a close resemblance between the redocked ligand conformations and the co-crystallized ones, indicating the procedure's ability to accurately reproduce the original binding geometry.


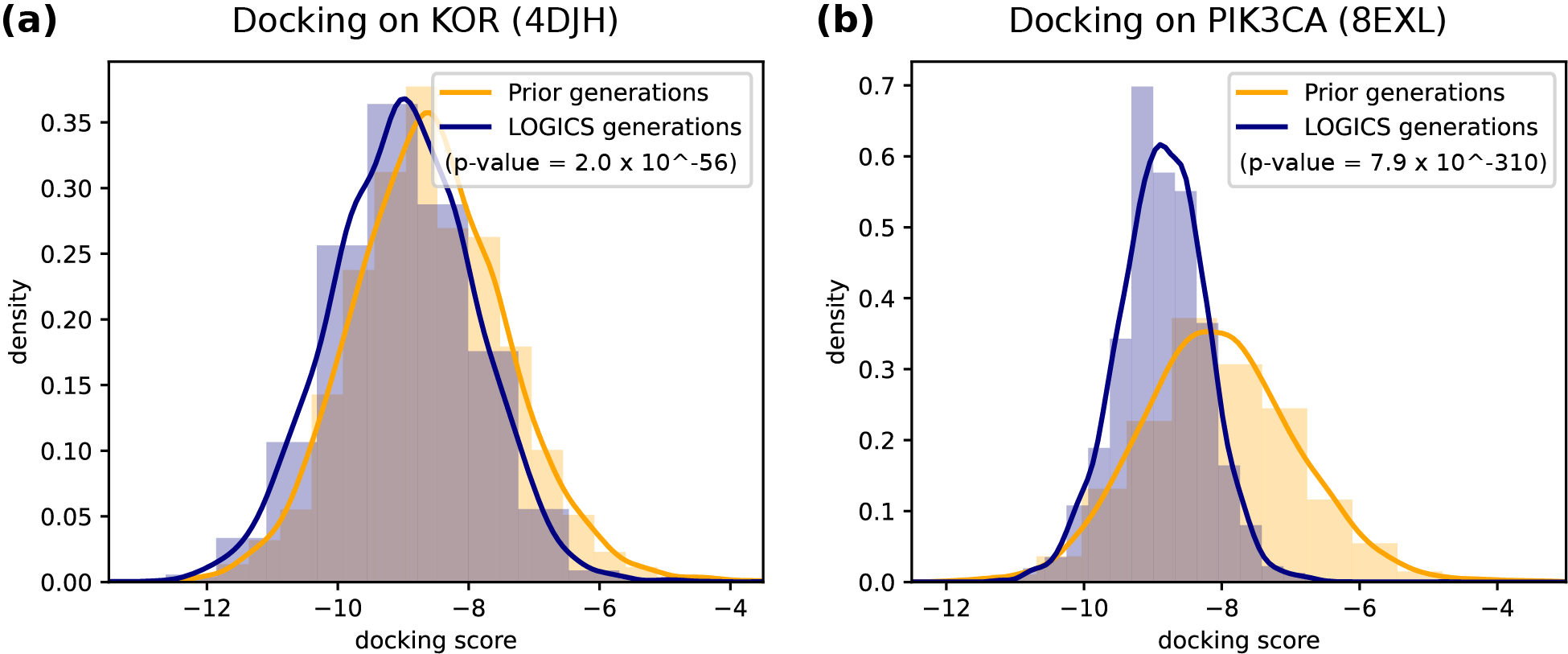


**Figure S8**. Density plots of docking scores (kcal/mol) on KOR (PDB ID: 4DJH) and PIK3CA (PDB ID: 8EXL) with generated compounds. (a), (b) Docking score distribution of 4,000 generations from the prior generator (orange) and LOGICS fine-tuned (blue) for KOR and PIK3CA, respectively. Two-tailed *t*-test between the two distributions is performed to evaluate the p-values.

The histogram and density plot of the docking scores are shown in **Figure S8**. In **Figure S8A** and **S8B**, optimized compounds show significantly stronger binding to the both targets compared to compounds from the prior generator (two-tailed *t*-test, KOR: p-value = 2.0 x 10^-56^, PIC3CA: p-value = 7.9 x 10^-310^). The difference between means of the prior and LOGICS scores was 0.413 kcal/mol and 0.820 kcal/mol for KOR and PIK3CA, respectively. For KOR, we note that the difference between the means is relatively marginal, which is supposedly attributed to the discrepancy in organisms, where we used Homo sapiens for the protein 3D structure, whereas the bioassay dataset had activities on KOR of Cavia porcellus.

## Section 16) Retrosynthetic prediction of generated compounds

To test the generated molecules in the wet lab, it is essential to know how to synthesize the new structures. In **Figure S9**, we have performed a retrosynthetic analysis on the generated molecules from **Figure 6**. The synthetic routes in **Figure S9** are predicted by AiZynthFinder software [54] with default parameters where the building block set is from ZINC database [55] and the reaction templates are from USPTO data [56]. The first synthetic route to be found in the search is reported for each molecule. The generated molecules for KOR activity in **Figure S9A** and **S9B** can be synthesized with 3 and 2 available building blocks, 4 and 1 reaction steps, respectively. The generated molecules for PIK3CA activity in **Figure S9C** and **S9D** can be synthesized with 4 building blocks for each, and 3 reaction steps for each. Corresponding ZINC ID for each building block in **Figure S9** is also specified.


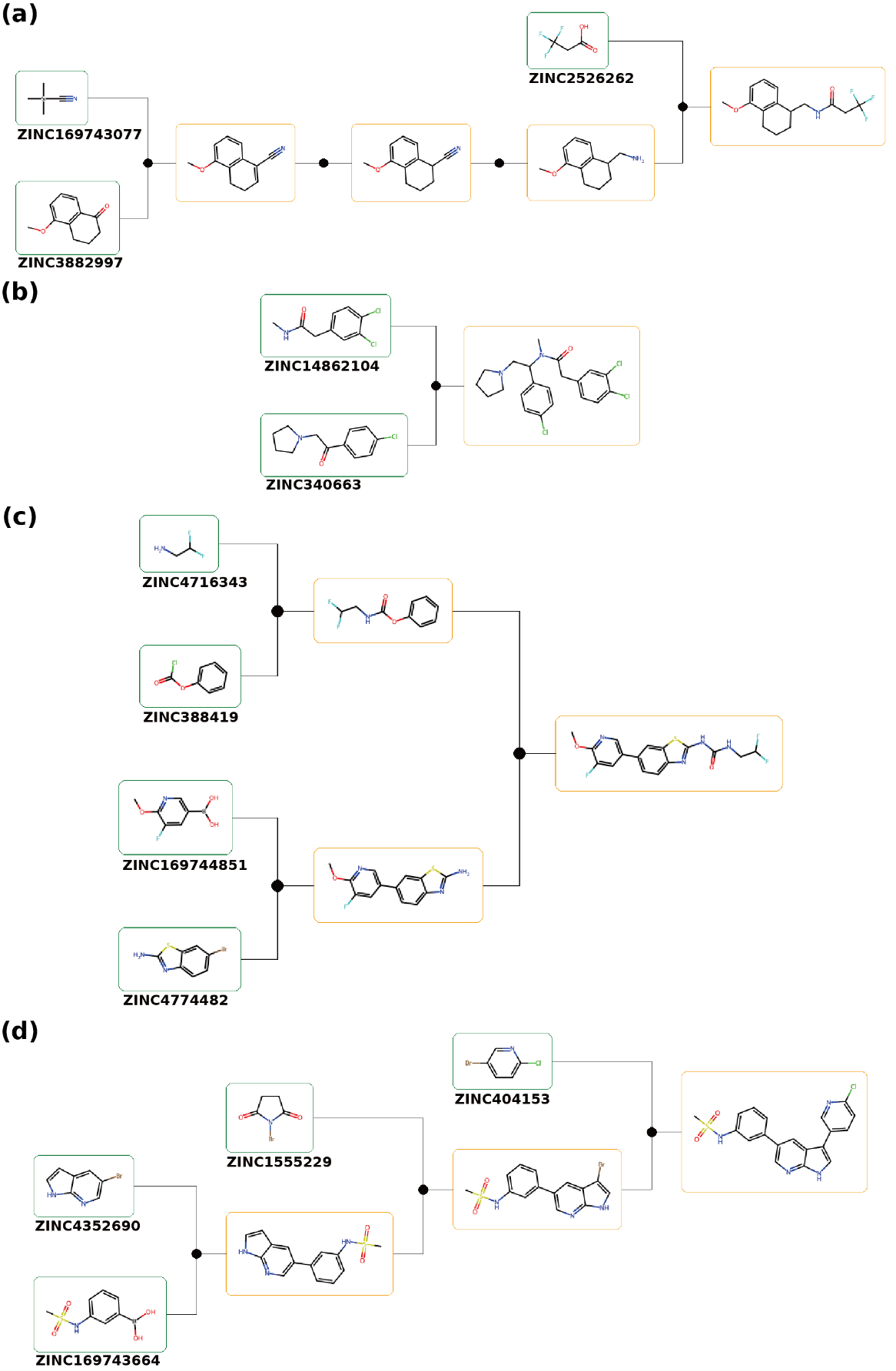


**Figure S9**. Retrosynthetic prediction of the generated molecules shown in **Figure 6**. The synthetic routes for (a), (b) generated molecules optimized for KOR activity, (c), (d) generated molecules optimized for PIK3CA activity are predicted by the AiZynthFinder software. Final and intermediate products are represented by orange nodes, and the building blocks are depicted by green nodes. The reactions used in each step are specified within the dotted rectangles. The default parameters are used in AiZynthFinder where the building block set is from the ZINC database and the reaction templates are from USPTO.

# References

1. Arus-Pous J, Blaschke T, Ulander S, Reymond JL, Chen H, Engkvist O: **Exploring the GDB-13 chemical space using deep generative models**. *J Cheminform* 2019, **11**(1):20.

2. Arus-Pous J, Johansson SV, Prykhodko O, Bjerrum EJ, Tyrchan C, Reymond JL, Chen H, Engkvist O: **Randomized SMILES strings improve the quality of molecular generative models**. *J Cheminform* 2019, **11**(1):71.

3. Skinnider MA, Stacey RG, Wishart DS, Foster LJ: **Chemical language models enable navigation in sparsely populated chemical space**. *Nat Mach Intell* 2021, **3**(9):759-770.

4. Devi RV, Sathya SS, Coumar MS: **Evolutionary algorithms for de novo drug design - A survey**. *Appl Soft Comput* 2015, **27**:543-552.

5. Yoshikawa N, Terayama K, Sumita M, Homma T, Oono K, Tsuda K: **Population-based De Novo Molecule Generation, Using Grammatical Evolution**. *Chem Lett* 2018, **47**(11):1431-1434.

6. Jensen JH: **A graph-based genetic algorithm and generative model/Monte Carlo tree search for the exploration of chemical space**. *Chem Sci* 2019, **10**(12):3567-3572.

7. Leguy J, Cauchy T, Glavatskikh M, Duval B, Da Mota B: **EvoMol: a flexible and interpretable evolutionary algorithm for unbiased de novo molecular generation**. *J Cheminform* 2020, **12**(1):55.

8. Zhou Z, Kearnes S, Li L, Zare RN, Riley P: **Optimization of Molecules via Deep Reinforcement Learning**. *Sci Rep* 2019, **9**(1):10752.

9. Stahl N, Falkman G, Karlsson A, Mathiason G, Bostrom J: **Deep Reinforcement Learning for Multiparameter Optimization in de novo Drug Design**. *J Chem Inf Model* 2019, **59**(7):3166-3176.

10. Chen Z, Min MR, Parthasarathy S, Ning X: **A Deep Generative Model for Molecule Optimization via One Fragment Modification**. *Nat Mach Intell* 2021, **3**(12):1040-1049.

11. Gomez-Bombarelli R, Wei JN, Duvenaud D, Hernandez-Lobato JM, Sanchez-Lengeling B, Sheberla D, Aguilera-Iparraguirre J, Hirzel TD, Adams RP, Aspuru-Guzik A: **Automatic Chemical Design Using a Data-Driven Continuous Representation of Molecules**. *ACS Cent Sci* 2018, **4**(2):268-276.

12. Kusner MJ, Paige B, Hernández-Lobato JM: **Grammar Variational Autoencoder**. In: *Proceedings of the 34th International Conference on Machine Learning; Proceedings of Machine Learning Research*: Edited by Doina P, Yee Whye T. PMLR 2017: 1945--1954.

13. Jin W, Barzilay R, Jaakkola T: **Junction Tree Variational Autoencoder for Molecular Graph Generation**. In*.*; 2018: arXiv:1802.04364.

14. Lim J, Ryu S, Kim JW, Kim WY: **Molecular generative model based on conditional variational autoencoder for de novo molecular design**. *J Cheminform* 2018, **10**(1):31.

15. Winter R, Montanari F, Steffen A, Briem H, Noe F, Clevert DA: **Efficient multi-objective molecular optimization in a continuous latent space**. *Chem Sci* 2019, **10**(34):8016-8024.

16. Hong SH, Ryu S, Lim J, Kim WY: **Molecular Generative Model Based on an Adversarially Regularized Autoencoder**. *J Chem Inf Model* 2020, **60**(1):29-36.

17. Olivecrona M, Blaschke T, Engkvist O, Chen H: **Molecular de-novo design through deep reinforcement learning**. *J Cheminform* 2017, **9**(1):48.

18. Merk D, Grisoni F, Friedrich L, Schneider G: **Tuning artificial intelligence on the de novo design of natural-product-inspired retinoid X receptor modulators**. *Commun Chem* 2018, **1**.

19. Popova M, Isayev O, Tropsha A: **Deep reinforcement learning for de novo drug design**. *Sci Adv* 2018, **4**(7):eaap7885.

20. Segler MHS, Kogej T, Tyrchan C, Waller MP: **Generating Focused Molecule Libraries for Drug Discovery with Recurrent Neural Networks**. *ACS Cent Sci* 2018, **4**(1):120-131.

21. Awale M, Sirockin F, Stiefl N, Reymond JL: **Drug Analogs from Fragment-Based Long Short-Term Memory Generative Neural Networks**. *J Chem Inf Model* 2019, **59**(4):1347-1356.

22. Liu X, Ye K, van Vlijmen HWT, AP IJ, van Westen GJP: **An exploration strategy improves the diversity of de novo ligands using deep reinforcement learning: a case for the adenosine A2A receptor**. *J Cheminform* 2019, **11**(1):35.

23. Zheng SJ, Yan X, Gu Q, Yang YD, Du YF, Lu YT, Xu J: **QBMG: quasi-biogenic molecule generator with deep recurrent neural network**. *J Cheminform* 2019, **11**.

24. Renz P, Van Rompaey D, Wegner JK, Hochreiter S, Klambauer G: **On failure modes in molecule generation and optimization**. *Drug Discov Today Technol* 2019, **32-33**:55-63.

25. Ahn S, Kim J, Lee H, Shin J: **Guiding Deep Molecular Optimization with Genetic Exploration**. In*.*; 2020: arXiv:2007.04897.

26. Papadopoulos K, Giblin KA, Janet JP, Patronov A, Engkvist O: **De novo design with deep generative models based on 3D similarity scoring**. *Bioorg Med Chem* 2021, **44**:116308.

27. Pereira T, Abbasi M, Ribeiro B, Arrais JP: **Diversity oriented Deep Reinforcement Learning for targeted molecule generation**. *J Cheminform* 2021, **13**(1):21.

28. Kerstjens A, De Winter H: **LEADD: Lamarckian evolutionary algorithm for de novo drug design**. *J Cheminform* 2022, **14**(1):3.

29. Mnih V, Kavukcuoglu K, Silver D, Graves A, Antonoglou I, Wierstra D, Riedmiller M: **Playing Atari with Deep Reinforcement Learning**. In*.*; 2013: arXiv:1312.5602.

30. Erikawa D, Yasuo N, Sekijima M: **MERMAID: an open source automated hit-to-lead method based on deep reinforcement learning**. *J Cheminform* 2021, **13**(1):94.

31. Ahmed A, Mam B, Sowdhamini R: **DEELIG: A Deep Learning Approach to Predict Protein-Ligand Binding Affinity**. *Bioinform Biol Insights* 2021, **15**:11779322211030364.

32. Gu Y, Zhang X, Xu A, Chen W, Liu K, Wu L, Mo S, Hu Y, Liu M, Luo Q: **Protein-ligand binding affinity prediction with edge awareness and supervised attention**. *Iscience* 2023, **26**(1):105892.

33. Kotsias PC, Arus-Pous J, Chen HM, Engkvist O, Tyrchan C, Bjerrum EJ: **Direct steering of de novo molecular generation with descriptor conditional recurrent neural networks**. *Nat Mach Intell* 2020, **2**(5):254-265.

34. Blaschke T, Engkvist O, Bajorath J, Chen H: **Memory-assisted reinforcement learning for diverse molecular de novo design**. *J Cheminform* 2020, **12**(1):68.

35. Pedregosa F, Varoquaux G, Gramfort A, Michel V, Thirion B, Grisel O, Blondel M, Prettenhofer P, Weiss R, Dubourg V *et al*: **Scikit-learn: Machine Learning in Python**. *J Mach Learn Res* 2011, **12**(null):2825–2830.

36. Paszke A, Gross S, Massa F, Lerer A, Bradbury J, Chanan G, Killeen T, Lin Z, Gimelshein N, Antiga L *et al*: **PyTorch: An Imperative Style, High-Performance Deep Learning Library**. In*.*; 2019: arXiv:1912.01703.

37. Brown N, Fiscato M, Segler MHS, Vaucher AC: **GuacaMol: Benchmarking Models for de Novo Molecular Design**. *J Chem Inf Model* 2019, **59**(3):1096-1108.

38. Polykovskiy D, Zhebrak A, Sanchez-Lengeling B, Golovanov S, Tatanov O, Belyaev S, Kurbanov R, Artamonov A, Aladinskiy V, Veselov M *et al*: **Molecular Sets (MOSES): A Benchmarking Platform for Molecular Generation Models**. *Front Pharmacol* 2020, **11**:565644.

39. Preuer K, Renz P, Unterthiner T, Hochreiter S, Klambauer G: **Frechet ChemNet Distance: A Metric for Generative Models for Molecules in Drug Discovery**. *J Chem Inf Model* 2018, **58**(9):1736-1741.

40. Heusel M, Ramsauer H, Unterthiner T, Nessler B, Hochreiter S: **GANs Trained by a Two Time-Scale Update Rule Converge to a Local Nash Equilibrium**. In: *Advances in Neural Information Processing Systems: 2017*: Edited by Guyon I, Luxburg UV, Bengio S, Wallach H, Fergus R, Vishwanathan S, Garnett R. Curran Associates, Inc. 2017.

41. Benny Y, Galanti T, Benaim S, Wolf L: **Evaluation Metrics for Conditional Image Generation**. *Int J Comput Vis* 2021, **129**(5):1712-1731.

42. Peyré G, Cuturi M: **Computational Optimal Transport**. In*.*; 2018: arXiv:1803.00567.

43. Solomon J: **Optimal Transport on Discrete Domains**. In*.*; 2018: arXiv:1801.07745.

44. Burkard RE, Çela E: **Linear Assignment Problems and Extensions**. In: *Handbook of Combinatorial Optimization: Supplement Volume A.* Edited by Du D-Z, Pardalos PM. Boston, MA: Springer US; 1999: 75-149.

45. Jonker R, Volgenant A: **A shortest augmenting path algorithm for dense and sparse linear assignment problems**. *Computing* 1987, **38**(4):325-340.

46. Virtanen P, Gommers R, Oliphant TE, Haberland M, Reddy T, Cournapeau D, Burovski E, Peterson P, Weckesser W, Bright J *et al*: **SciPy 1.0: fundamental algorithms for scientific computing in Python**. *Nat Methods* 2020, **17**(3):261-272.

47. Neil D, Segler MHS, Guasch L, Ahmed M, Plumbley D, Sellwood M, Brown N: **Exploring Deep Recurrent Models with Reinforcement Learning for Molecule Design**. In: *International Conference on Learning Representations: 2018*.

48. Thomas M, O'Boyle NM, Bender A, de Graaf C: **Augmented Hill-Climb increases reinforcement learning efficiency for language-based de novo molecule generation**. *J Cheminform* 2022, **14**(1):68.

49. Guo J, Schwaller P: **Augmented Memory: Capitalizing on Experience Replay to Accelerate De Novo Molecular Design**. In*.*; 2023: arXiv:2305.16160.

50. Ramsundar B, Eastman P, Walters P, Pande V, Leswing K, Wu Z: **Deep Learning for the Life Sciences**: O'Reilly Media; 2019.

51. Alhossary A, Handoko SD, Mu Y, Kwoh CK: **Fast, accurate, and reliable molecular docking with QuickVina 2**. *Bioinformatics* 2015, **31**(13):2214-2216.

52. Trott O, Olson AJ: **AutoDock Vina: improving the speed and accuracy of docking with a new scoring function, efficient optimization, and multithreading**. *J Comput Chem* 2010, **31**(2):455-461.

53. O'Boyle NM, Banck M, James CA, Morley C, Vandermeersch T, Hutchison GR: **Open Babel: An open chemical toolbox**. *J Cheminform* 2011, **3**:33.

54. Genheden S, Thakkar A, Chadimova V, Reymond JL, Engkvist O, Bjerrum E: **AiZynthFinder: a fast, robust and flexible open-source software for retrosynthetic planning**. *J Cheminform* 2020, **12**(1):70.

55. Irwin JJ, Tang KG, Young J, Dandarchuluun C, Wong BR, Khurelbaatar M, Moroz YS, Mayfield J, Sayle RA: **ZINC20-A Free Ultralarge-Scale Chemical Database for Ligand Discovery**. *J Chem Inf Model* 2020, **60**(12):6065-6073.

56. Thakkar A, Kogej T, Reymond J-L, Engkvist O, Bjerrum EJ: **Datasets and their influence on the development of computer assisted synthesis planning tools in the pharmaceutical domain**. *Chemical Science* 2020, **11**(1):154-168.
